# Sanitation and marriage markets in India: Evidence from the Total Sanitation Campaign^☆^

**DOI:** 10.1016/j.jdeveco.2023.103092

**Published:** 2023-06

**Authors:** Britta Augsburg, Juan P. Baquero, Sanghmitra Gautam, Paul Rodriguez-Lesmes

**Affiliations:** a Institute for Fiscal Studies, United Kingdom; b Boston College, United States of America; c Department of Economics, Washington University in St Louis, United States of America; d School of Economics, Universidad del Rosario, Colombia

**Keywords:** Matching, Intra-household, Marriage markets, Sanitation, Sorting

## Abstract

This paper measures the additional value of sanitation within the marriage arrangement. We use data from the Indian human development household survey (IHDS) to model the marital decisions of men and women in rural India and to estimate the marital surplus (the gains from being married). We use the model to demonstrate that the government’s Total Sanitation Campaign (TSC) increased marital surplus and changed marriage market outcomes for men and women. Decomposition reveals (i) that sanitation makes it more attractive to be in a marriage for both gender, and (ii) that TSC exposure led to a decrease in the wife’s surplus share, implying a redistribution of gains within the marriage.

## Highlights

• Promoting household public goods can alter marriage market outcomes.
• We show this to be the case for India’s Total Sanitation Campaign (TSC).
• The TSC made being married more attractive, increasing marital surplus.
• While the increase benefits both genders, the wife’s surplus share decreases.
• Redistribution of marital gains are important for policy makers to consider.

## 1 Introduction

Many social programs and policy interventions are expected to have greater benefits for one gender than the other, so they focus their promotional effort on a particular gender. For example, a strong female focus is evident in the promotion of household public goods such as cooking stoves (Miller and Mobarak, 2013) or in the context of water sanitation and hygiene (Stopnitzky, 2017). However, despite a general acceptance that a female-focused approach is justified for these goods, impact evaluations have generally focused on household-level outcome indicators. Very little is known about women specific impacts and – the focus of this paper – impacts on marriage market outcomes. If promoted goods are indeed of public benefit within a household, prospective spouses might consider them in their marriage decision, implying a potential change to the benefits of marriage and hence marriage market outcomes. In this paper, we focus on quantifying the added marital value of sanitation, whether it entails a gain or a loss on the marriage market, and, if so, for whom. In doing so, we provide the first quantitative evidence that gender-focused sanitation interventions can indeed change marriage market outcomes.^1^

Our objective to quantify the added marital gain from sanitation necessarily intersects with two fundamental questions in the study of marriage markets: “Who marries whom?” and “How does the market clear?” This interlinkage poses notable identification challenges which arise from sanitation preferences being correlated with observable (e.g., wealth) and unobservable (e.g., marital preferences) characteristics that are relevant for matching decisions. Moreover, in this case, the division of the gains is endogenous and determined in the marriage market equilibrium. We use a structural approach to provide empirical evidence in support of our argument. A central contribution of this paper is to explicitly model the marriage decisions of men and women in order to decompose the total effect of sanitation programs — in our case, the Government of India (GoI)’s Total Sanitation Campaign (TSC). The TSC was a nationwide program to increase household-level sanitation and focused primarily on the provision of financial incentives. Like sanitation interventions more generally, it considered women to be an important target group as homemakers and caregivers for children, the sick, and the elderly (Cavill et al., 2018, Radtke, 2018).

It is by now well established that the TSC and TSC-like interventions can have positive impacts on household sanitation take-up (Barnard et al., 2013, Clasen et al., 2014, Hammer and Spears, 2016, Patil et al., 2014, Pattanayak et al., 2009, Arnold et al., 2010, Stopnitzky, 2017). The first step in our analysis is to estimate the total effect of the TSC program, focusing on households that are active in the marriage market. By doing so, we provide a generalization of findings by Stopnitzky (2017), who evaluates the TSC as implemented in one Indian state, Haryana. The government of Haryana took a distinct approach by explicitly linking its promotion of sanitation to the marriage market in its “No Toilet No Bride” (NTNB) campaign. The advertisement campaign promotes a narrative that encourages families with marriage-age girls to ask prospective suitors to provide and, if necessary, construct a latrine in the bride’s new home — thereby including sanitation in the prospective couple’s marital living arrangement. Using three data sources: the Indian Human Development Survey (IHDS), 2001 and 2011 census data, and TSC performance monitoring data (WSP, 2011). Exploiting district and time variation in TSC exposure and multiple observations of the marriage market, we find that the TSC led to a significant increase in sanitation ownership among households active in the marriage market, particularly when the prospective spouse in the household is male. These households are 6.1 percentage points (ppts) more likely to own a toilet, which constitutes a 15% increase in household sanitation coverage. Moreover, we find that the TSC impact is larger in marriage markets where women are relatively scarce. We corroborate these findings by linking take-up responses to changes in the marriage patterns observed in the data.

While these results provide indirect evidence that having sanitation adds marital value, they deliver an incomplete assessment of the overall effect of the TSC. This is because the reduced form results do not consider equilibrium effects such as the availability of spousal alternatives and changes in matching opportunities in the market that result from the increase in the number of households with sanitation.

We develop and estimate a structural model that allows us to decompose and quantify this overall policy impact on marital sorting, marital surplus, and the division of that surplus among partners, thereby identifying the overall importance of sanitation in the marriage market. Our model of the marriage market uses a simple matching framework under transferable utility à la Choo and Siow (2006), in which prospective spouses match based on their wealth type. To allow for changes in the composition of who marries whom, and for differential unobserved marital preference, men and women simultaneously choose their spouse *and* whether the new living arrangement includes sanitation or not. In other words, prospective spouses decide not only whether to marry, and whom to marry, but also the type of living arrangement they want to share. Allowing for different types of living arrangements delivers an empirically tractable marital surplus function that permits sorting based on unobserved tastes for sanitation. We extend the framework to multiple markets using districts, and exploit variation over time (before and after TSC exposure) to achieve model identification.

Our estimates provide interesting insights into the determinants of the marital surplus that drive individuals to sort into marriage relative to remaining single. We show that the marital surplus from sanitation increases with the wealth of the household and varies with the wealth of the couple. This suggests that taste and household demand for sanitation vary with the couple’s wealth level. In other words, having sanitation in the marriage is not universally more attractive than an arrangement without. We then use the estimated model to conduct a structural policy evaluation of the TSC to assess whether the policy alters the marital surplus and the division of the surplus.

The evaluation highlights three main findings. First, we document a significant increase in the marital surplus due to TSC exposure. In other words, a decrease in the price of sanitation, a household public good, increases the gains from the marriage that a couple receives relative to singlehood. This increase in marital surplus is consistent with the change in marriage behavior — having sanitation makes it more attractive to be in a marriage for both men and women, inducing some to marry. The increased pool of potential partners leads to changes in sorting patterns. Second, the change in marital sorting results in a redistribution of gains across matched types, namely a higher marital match surplus for couples where men are wealthier than women and a lower marital match surplus for most remaining cases. Third, the policy also reallocated gains within a marriage, where some of the gains in toilet accessibility were redistributed to men, away from women, through the marriage equilibrium process. It is important to note that our results do not imply that the women are necessarily worse off ex-post TSC exposure. The total marital utility is higher, and so in equilibrium, we see more marriages and, thus, the entry of men and women. However, the division of this additional marital utility is not necessarily equal among partners, which is demonstrated by analyzing TSC’s impact on the wife’s surplus share.

To the best of our knowledge, we are the first to explicitly model sanitation’s importance in the marriage market. Stopnitzky (2017), who evaluates “No Toilet, No Bride”, relies on a triple-difference specification, comparing the sanitation status of households with and without boys of marriageable age, living in the policy-implementing state or a comparable northern Indian state, before and after program implementation. Analyzing heterogeneity in impacts by the scarcity of women, he infers that impacts are driven by markets where women are scarce. However, instead of modeling the marriage market equilibrium, the paper relies on an empirical proxy, i.e., sex ratios, in order to draw inferences regarding marriage market outcomes from sanitation take-up regressions. The use of sex ratios as an empirical proxy for marriage market conditions is common in the literature (e.g., Abramitzky et al. (2011), Angrist (2002), Charles and Luoh (2010), and Gupta (2014)). Nevertheless, the use of sex ratios in marriage rate regressions has a key limitation: it ignores the availability of alternatives. By allowing for spousal alternatives, the Choo and Siow (2006) marriage matching function encapsulates both the equilibrium and the heterogeneous policy effect, thereby capturing the overall equilibrium market response. Our results show that the marriage market equilibrium determines the subsequent division of the surplus in marriage. Therefore, scarcity per se does not imply that female-targeted programs necessarily result in better outcomes for women.

This paper also contributes to the sanitation policy literature in two important ways. First, we show that marriage markets are an important and relevant channel through which the impact of the TSC mediates. On this, our paper marks a considerable departure from the literature that has emphasized the importance of gender yet has largely ignored the role of marriage markets. Our results illustrate that policies that target household incentives for public goods affect the economic benefits of marriage, which in turn affect marriage behavior and the division of the surplus. Second, we add to the current policy discussion by showing that the policy implications change once we account for marriage market effects. Much of the literature exploring sanitation and gender has focused on the impact of shifts in the decision weights or ‘bargaining power’ on demand for sanitation within the marriage. In this case, the decision weight determining the surplus’s division is treated as fixed or policy invariant. Under this scenario, policy prescriptions such as the NTNB program can be used to increase the overall demand for sanitation. In contrast, our paper shows that the division of the surplus is an equilibrium object determined in the marriage market and, as such, not policy invariant. In this scenario, we show that although policies such as the TSC increased gains from marriage, they also implied a reduction in the female surplus share, which policymakers should consider. Overall, we argue that a careful analysis of the marriage market, as in this paper, is an integral component of the overall policy impact on existing and future marriages, and is important to infer the determinants of men’s and women’s well-being.^2^

The rest of the article is organized as follows. Section 2 describes the TSC policy, data, and features of the Indian marriage market. Section 3 presents the effect of the policy on sanitation take-up among households active in the marriage market and describes the marital sorting patterns observed in our sample. The theoretical framework is developed and estimated in Section 4. Our main empirical findings are presented and discussed in Section 5. Finally, Section 6 concludes.

## 2 The context

### 2.1 The Total Sanitation Campaign

In 1999, after 13 years of relatively poor performance of the Centrally Sponsored Rural Sanitation Program (CRSP), the GoI created the Total Sanitation Campaign (TSC), which set the stage for India’s rural sanitation policy up to 2011.^3^ Its aim was to accelerate sanitation coverage in rural areas by providing access to toilets among the entire population by 2012.

Two of the key insights that informed TSC design were that toilet construction and usage do not necessarily go hand in hand, and, therefore, the benefits of sanitation needed to be made clearer to households. To this end, the policy advocated for communities to lead their own sanitation improvements with heavy involvement from GP leaders and community groups, and placed a strong emphasis on information, education, and communication (IEC) at various levels: in communities, with Gram Panchayat (GP) institutions,^4^ ${}^{,}$^5^ and with students in schools and rural childcare centers (Anganwadi). Financial incentives then continued to be implemented, focusing on below poverty line (BPL) households and post-construction. The policy also provided a range of technology options and developed a supply chain to meet the demand (for various models) stimulated at the community level.

**Fig. 1:**
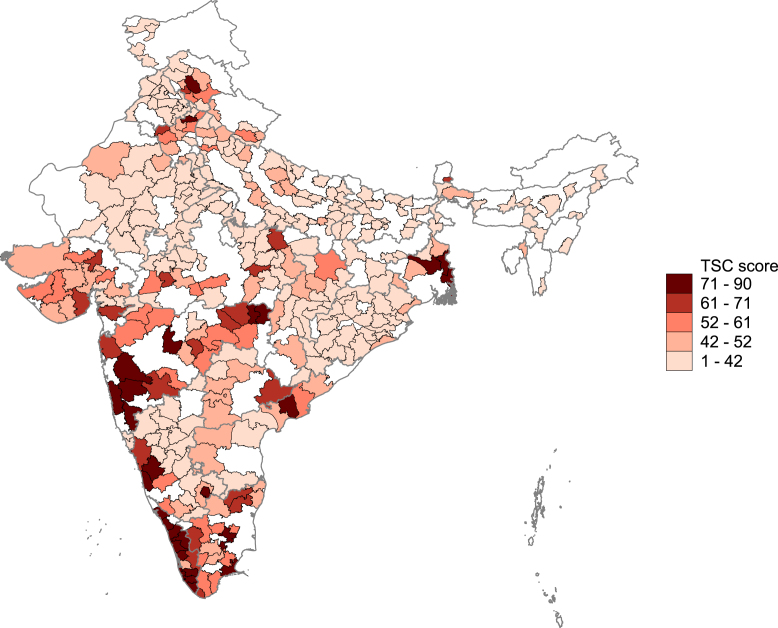
Grand Score TSC performance. *Note*. Own calculations using data from (WSP, 2011). The figure shows the Grand Score TSC implementation performance measure by district. The “Grand Score” is an index that measures performance based on eight key TSC performance indicators, covering inputs, outputs, processes, and outcomes, where each indicator is allocated a maximum score. Scores add up to a maximum of 100 and are available at the district level.

A strong upward trend in rural sanitation coverage has been documented over the course of program implementation. IHDS data indicates that in 2004 48% of households owned a toilet, which increased to 57% in 2011. Several studies, including randomized control trials, show that the TSC was a significant driving force behind this increase (Barnard et al., 2013, Clasen et al., 2014, Hammer and Spears, 2016, Patil et al., 2014, Pattanayak et al., 2009, Stopnitzky, 2017), establishing its effectiveness in increasing toilet ownership, with impacts that are said to be high compared with many other evaluated sanitation policies (Garn et al., 2017).^6^ At the same time, significant disparities across states and districts have been documented. WSP (2011) illustrates the unequal performance of TSC among Indian districts through a global monitoring index (“the Grand Score”), as shown in Fig. 1. The Grand Score measures performance according to eight key indicators that capture inputs, outputs, processes, and outcomes.^7^ In our analysis, we define districts highly exposed to the TSC policy as those that receive a score of 61 or above, which applied to a total of 54 districts (16% of the IHDS sample). We will refer to them in the remainder of the paper as “high-exposure” or “high” TSC districts (as compared with “low-exposure” or “low” TSC districts). On the map, these high TSC districts are shaded in the two darkest colors. Acknowledging that this cut-off (while chosen to capture the long right tail of the distribution) is to an extent arbitrary, we will conduct robustness checks around this choice in our analysis.

In our analysis, we focus on districts for which we have a Grand Score and which are included in the Indian Human Development Survey (IHDS). The IHDS is a household-level panel that provides rich micro-data on household composition, including individual demographics and socio-economic indicators, located in 375 of India’s 741 districts. We use two waves, collected in 2004 (Desai et al., 2010) and 2011 (Desai et al., 2018). Our final analysis sample covers 345 districts.^8^

### 2.2 The Indian marriage market

A number of distinctive features of the Indian marriage market are complementary to our analysis. For one, the convention of marriages taking place within a specified physical distance provides us with non-overlapping and multiple observations. Second, highly prevalent assortative matching on observable characteristics, including wealth, allows us to quantify the added value from sanitation vis- à-vis other marital assets. We further describe other important features of the market that determine our sample, which we control for in our analysis and exploit for heterogeneity analysis.

#### 2.2.1 Presence of multiple marriage markets

The Indian marriage market is characterized by the practice of patrilocal or virilocal residence, which almost always dictates that a bride will move from her natal home to her groom’s home (Desai and Andrist, 2010).^9^ In line with this, we see in the IHDS that no more than 12% of women grew up in the same village as their husbands. Importantly though, spouses are chosen from geographically close areas — the couples’ villages of origin are, on average, a distance of 3 h away with locally common transportation (see Appendix Table A1). Only 4% of total female migration (including for education and other non-marriage purposes) is inter-state, while 9.8% is inter-district (but intra-state) (Kone et al., 2018), implying that the vast majority of brides’ destinations are intra-district. This feature, as most recently highlighted for the Indian context by Beauchamp et al. (2017), provides us with not just one but multiple distinct marriage markets for our analysis and generates overidentifying restrictions on the model parameters.

#### 2.2.2 Assortative matching by wealth

A second important feature of the Indian marriage market is the strong pattern of positive assortative mating observed in the matching process. We focus on wealth because economic indicators, such as land and income, have been shown to be important factors in the decision regarding whom to marry in the Indian context (Rao, 1993, Borker et al., 2019).^10^

**Fig. 2:**
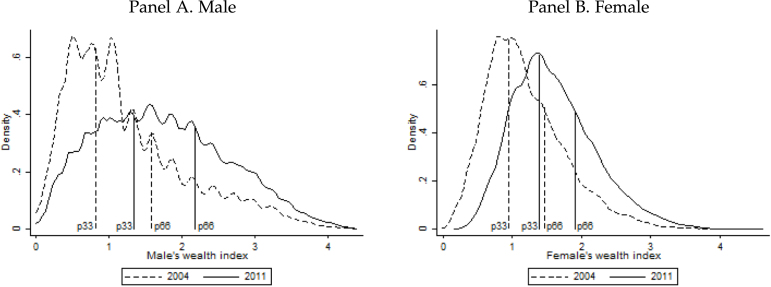
Wealth index distribution. *Note.* Own calculations using data from the IHDS waves 2004 and 2011. The figure shows the wealth index distribution by gender and over time. The wealth index, defined at household level, is constructed using a principal component analysis of self-reported assets in the household, which include bicycle, sewing machine, generator set, grinder, motorcycle, TV, air cooler, clock, electric fan, chair/table, cot, telephone, cell phone, refrigerator, pressure cooker, car, air conditioner, washing machine, computer, credit card. For males it corresponds to the household information, while for females it is the prediction based on observed characteristics: age, education, literacy, English knowledge, caste and state. The prediction comes from a model for single women where the dependent variable is the wealth index of their family.

Our proxy for wealth is based on self-reported asset ownership using principal component analysis (PCA),^11^ but we will adopt different approaches to construct pre-marital wealth for the wife and the husband. The almost universal practice of patrilocal exogamy, i.e., the bride moves into the groom’s family’s house, implies that we can proxy the groom’s pre-marital wealth by using the information on asset ownership as reported for the household in which the couple resides. For the bride’s pre-marital wealth, we instead use a predicted asset score that is based on how observable characteristics of single women (age, education, literacy, English knowledge, caste, and state) predict the asset index of their family. Fig. 2 shows the distribution of the asset index by year for both genders. In line with significant economic growth over this period, we observe a rightward shift in the distribution between 2004 and 2011. In our model, we use a discrete version of these variables based on the terciles defined over gender and year. The vertical lines in Fig. 2 indicate the limits of these categories.

We calculate the Pearson’s correlation coefficients to characterize the degree of marital matching by wealth. We find a correlation coefficient between the groom’s and bride’s wealth index of 0.679 in 2004 and 0.676 in 2011. A value of 1 would indicate a perfect correlation, so these statistics confirm a high degree of matching by wealth in the Indian context. If we consider instead the discrete variable (low, medium, high wealth), Kruskal’s Gamma correlation coefficients produce similar results: a rank correlation of 0.736 in 2004/05 and 0.724 in 2011.^12^

In our analysis, we take into account that wealth and sanitation ownership tend to co-vary. The correlation coefficient of 0.49 in 2004 shows that there is considerable heterogeneity in sanitation ownership at different levels of wealth. It is interesting to note that the coefficient of 0.49 is lower than the correlation of asset wealth with owning a color TV (0.76) or a refrigerator (0.74), but higher than for owning a pressure cooker (0.46).

#### 2.2.3 Other marriage market features shaping our analysis

***Caste and age.*** Our analysis will include a set of controls to ensure we account for additional characteristics known to shape the Indian marriage market.

First among these is the longstanding practice of caste endogamy, which dictates that marriages must take place within a particular social group, determined here by caste. Over the period 2004–2011, less than 6% of marriages took place between people belonging to different castes (Table A1). This figure has remained stable despite strong economic growth and is also similar across high and low TSC districts (Table A2). Banerjee et al. (2013) show that underlying this statistic is a strong preference and low cost to marry someone of the same caste, and recent genetic analysis has established that these patterns of endogamous marriage have been in place for over 2000 years (Moorjani et al., 2013). We observe a similar proportion of aggregate castes for low and high TSC districts (Table A2).

**Fig. 3:**
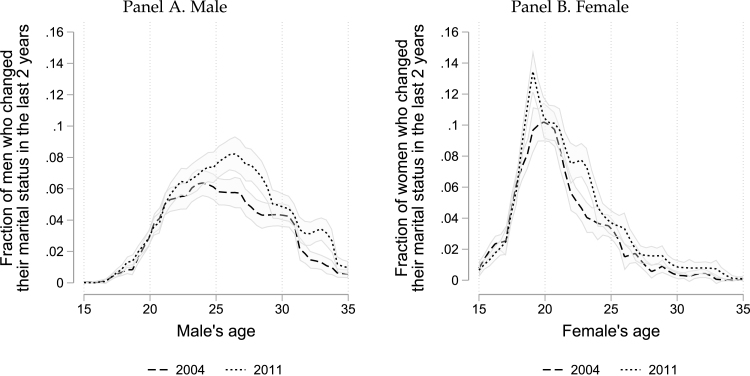
Change in marital status. *Note.* Own calculations using data from the IHDS waves 2004 and 2011. This figure shows the fraction of respondents who changed their marital status in the last two years by age, gender, and year of the survey. The sample includes all men and women aged 15 to 35 years. Change in marital status corresponds almost entirely to singles getting married, as almost no divorces or deaths of a spouse are observed. The measure is based on age at the time of the survey and the reported age of marriage. Confidence intervals for the local linear polynomials correspond to 95%.

Age, meanwhile, is another characteristic that has been explored in relation to marriage matches. The median age of brides was 18 years in 2011 (one year higher than in 2004, and in high TSC relative to low TSC districts, shown in Tables A1 and A2 respectively). By the time women reach their late 20 s, the likelihood of them changing their marital status approaches zero (Fig. 3).^13^ This is in line with the existence of unofficial age limits by which women are expected to be married (Jaggi, 2001), which is at least partially driven by parental perceptions that the quality of a match deteriorates quickly with a girl’s age at leaving school (Adams-Prassl and Andrew, 2019). Men get married at a later age, and their likelihood of marriage levels off when they reach their mid-30s.^14^ The fact that marriages are rarely reversed leads us to focus our analysis on households of all single and married females (males) aged 15 to 34 at the time of the survey, i.e., excluding those who are unlikely to be active in the market.

**Fig. 4:**
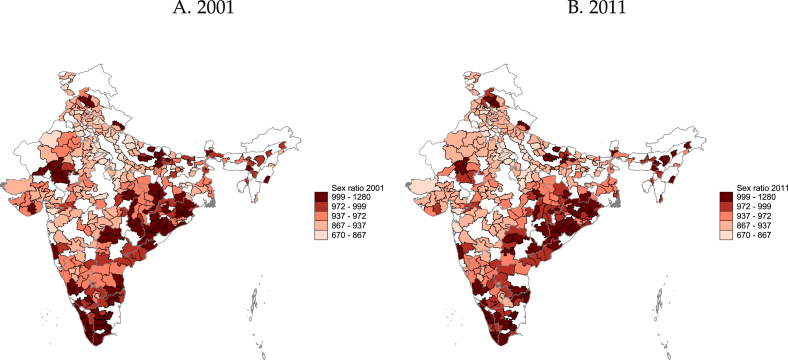
District-level sex ratio. *Note.* Own calculations using Indian census data, 2001 and 2011. Sample includes 15 to 34–year-olds.

***Sex ratio.*** Considerable attention has been paid to the fact that India is characterized by huge disparities in sex ratios across the country, ranging from equally balanced to only two women for every three men in the north of the country (Fig. 4). Research in contexts other than India suggest that such gender imbalances tend to lead to improved marriage outcomes for the scarcer sex (Abramitzky et al., 2011, Angrist, 2002, Charles and Luoh, 2010).^15^ We take sex ratios as a given market characteristic that varies across districts and analyze whether the differences across districts affect our findings. To do this, we conduct heterogeneity analysis by high and low district ratios. We define high sex ratio as a district where there are at least 999 women per 1000 men aged 15–34, which we found accounted for nearly 20% of the sample in both 2004 and 2011.^16^

## 3 Sanitation policy and the Indian marriage market

In this section, we analyze interactions between TSC performance and marriage market characteristics. To do this, we analyze differential sanitation uptake in high and low districts by sex ratio, focusing on households that are likely to be active in the marriage market. We further consider whether the high degree of matching we observed above differs by TSC performance. Any observed differences we present provide indirect evidence of TSC impacts on marriage market outcomes. These, however, do not take into account general equilibrium effects, which we will analyze in Section 4.

### 3.1 The TSC, sanitation uptake, and marriage market characteristics

As discussed above, previous literature has established that the TSC led households to invest in sanitation. Here, we explore whether households that are about to enter or are already active in the marriage market are particularly responsive to the TSC policy. We also attempt to determine whether they did so differently depending on underlying market conditions, as indicated by the district-level sex ratio. We use a repeated cross-section of all households where the household head has at least one son or daughter aged 15–34. We estimate the following difference-in-difference specification:

(1) $$Y_{idt}=\alpha_{0}+\alpha_{1}Post_{t}+\delta HighTSC_{d}+\gamma HighTSC_{d}\times Post_{t}+X_{idt}\Lambda +\eta_{d}+\epsilon_{idt}$$

where $Y_{idt}$ denotes an indicator that household i in district d at time t adopts sanitation. We define Post=1 for survey wave 2011 and Post=0 for 2004/05; $HighTSC_{d}=1$ for households located in a district characterized by high TSC performance, which corresponds to a Grand Score of at least 61, and 0 otherwise. $X_{idt}$ is a vector of household controls: age, gender, and marital status of the oldest household member 15–34 years old, as well as his/her household’s wealth index; household size, education level of the household head (no education [base], primary, incomplete secondary, secondary, and above secondary); caste of the household head (Brahmin [base], high caste, other backward castes, Dalit, Adivasi, Muslim, and Sikh or Jain); and an indicator variable for living in a rural zone. District-level fixed effects are given by $\eta_{d}$ and $\epsilon_{idt}$ corresponds to the unobserved component. The coefficient of interest is γ, indicating the impact of high TSC. In all regressions, standard errors are clustered at the district level.

#### 3.1.1 Parallel trends

Whether γ identifies the causal effect of high TSC performance on sanitation take-up depends on the validity of the parallel trends assumption that our analysis relies upon. We therefore assess whether, on average, sanitation ownership would have evolved along similar trends in districts where the TSC was implemented well and where implementation lagged, had the policy not been implemented.

**Fig. 5:**
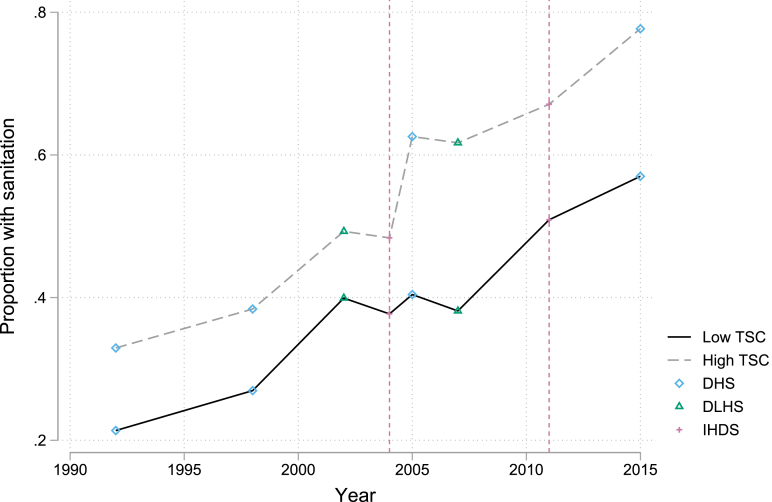
Sanitation coverage by TSC groups (state level). *Note.* This figure shows the proportion of household with sanitation at the state level by TSC exposure groups. High TSC corresponds to a mean Grand Score of at least 61, computed by averaging the Grand Scores of the districts using the IHDS 2004 household sampling weights. We compute the proportion of households who own a toilet using data from IHDS (2004 and 2011), the Indian DHS (Demographic and Health Survey) 1992–93, 1998–99, 2005–06, and 2014–15 rounds, and the India DLHS (District Level Health Survey) 2002–04 and 2007–08 rounds.

To examine the parallel trends assumption we, like Stopnitzky (2017), we must turn to additional data sets to assess this assumption. In Fig. 5, we combine information from four rounds of the Indian Demographic and Health Survey (DHS), two rounds of the District Level Household and Facility Survey (DLHS), and the IHDS waves. Plotting sanitation ownership rates by high and low TSC performance over time, the figure shows a steady increase in sanitation ownership prior to the introduction of the TSC, which on average develops similarly in high and low TSC districts. The trends diverge thereafter, though, particularly in the mid-2000s.

#### 3.1.2 Results on take-up

Table 1 shows the results, focusing on households where the oldest member within the marriage age range of 15–34 years is male. We find that for these households, toilet ownership increases by 6.1 percentage points (ppts) due to living in a high TSC district (column 1). This result is significant at the 10% level and robust to alternative definitions of female shortage and TSC exposure, as shown in Table A3 in the appendix. The percentage point increase is by chance the same as identified by Stopnitzky (2017), however, from a higher base and over a longer time period. The base in Haryana when the NTNB campaign was introduced was 29%, implying that over the four year-period analyzed by Stopnitzky (2017) was 21%. In our cross-India sample of high TSC districts, the base was 41%, and we document a 15% increase over this base over the 6-year period from 2004. For females, the estimated coefficient is smaller at 3.84 ppts and statistically insignificant (Table A4). The difference in the impact across gender is not statistically significant due to the large standard errors of the estimates (Table A5).

**Table 1:** TSC impact on sanitation for households with sons.

	Sanitation take-up		
	All	High sex ratio	Low sex ratio
	(1)	(2)	(3)
HighTSC×Post	0.061*	−0.047	0.132***
			

Observations	27 993	5784	22 209
Districts	344	100	279
$R^{2}$	0.480	0.516	0.476
Wald test (2) = (3): p-val		0.001	

*Note.* Own calculations using data from the IHDS waves 2004 (Post=0) and 2011 (Post=1). The table shows the TSC impact on sanitation for households with an oldest marriageable son. The sample consists of households of all single and married males aged 15 to 34 at the time of survey. HighTSC=1 corresponds to a grand score for implementation of at least 61, and HighTSC=0 otherwise. The Grand Score per district is taken from WSP (2011). The high sex ratio corresponds to districts with at least 999 women per 1000 men in the age range 15–34, while the low sex ratio is districts with less than 999. District level sex ratio information was computed using data from the population census 2001 and 2011. As controls, we use Post, the age and marital status of the individual for whom the household is in the sample; the wealth index of the household; household size; education level of the household head (no education [base], primary, incomplete secondary, secondary, and above secondary); caste (Brahmin [base], high caste, other backward caste, Dalit, Adivasi, Muslim, and Sikh or Jain); an indicator variable for the rural zone; and fixed effects at the district level. Standard errors, clustered at the district level, are shown in parentheses. A Wald test of equivalence of coefficients that was performed by jointly estimating coefficients for columns 2 and 3 in a single regression, interacting all variables (including controls) with a dummy indicating whether the observation corresponds to a high sex ratio district or not. Significance: *** p < 0.01, ** p < 0.05, * p < 0.1.

#### 3.1.3 Heterogeneity by marriage market characteristics

In addition to shedding light on whether households active in the marriage market respond to the TSC campaign, we consider heterogeneity in these impacts by underlying market conditions. Estimating impacts separately for districts characterized by high and low sex ratios (columns 2 and 3 of Table 1), we find that the average impact is purely driven by areas where women are scarce: in these districts, we find a 13.2 ppt increase in toilet ownership due to high TSC, which is significant at the 1% level and significantly different from the negative and insignificant coefficient in high sex ratio areas. For females, the coefficient is smaller at 8.6 ppts and significantly different from the male coefficient (Table A5), suggesting that households with a marriageable boy tend to react more acutely to the TSC campaign. These results are in line with Stopnitzky (2017), who found in the context of the “No Toilet No Bride” campaign in Haryana that households with men of marriageable age were more likely to take up sanitation when exposed to the campaign than households with women of similar ages.

In Table 2, we break down the analysis further, taking into account whether a household falls within a high- or low-wealth category based on the median of the wealth index distribution for each wave. We continue to find no impacts in high sex ratio districts. In low sex ratio districts, impacts are driven by households at the top of the wealth distribution. These high-wealth households with a son in line to get married are 18.9 ppts more likely to construct a toilet due to living in high TSC districts. For low-wealth households in low sex ratio districts, the estimated impact is also positive at 8.3 ppts and not distinguishable from the high-wealth estimate; however, it is itself insignificant.

An important takeaway from the analysis presented in this section is that the TSC program likely affected the marriage market equilibrium: the extent of uptake is a function of marriage market characteristics and attributes such as the scarcity of women and wealth. In addition, Table 1, Table 2 provide indirect evidence of the importance of incorporating heterogeneous policy effects of the TSC in our analysis of the marriage market equilibrium. We unpack this link in Section 5, based on the structural model presented in Section 4. Before doing so, we once more turn back to marital sorting.

**Table 2:** TSC impact on sanitation for households with sons by wealth and sex ratio.

	Sanitation take-up			
	High sex ratio		Low sex ratio	
	(1)	(2)	(3)	(4)
	High wealth	Low wealth	High wealth	Low wealth
HighTSC×Post	−0.028	−0.005	0.189***	0.083
				

Observations	3133	2646	11 660	10 542
$R^{2}$	0.432	0.401	0.365	0.386
Wald test p-val				
(1)=(2)	0.031			
(3)=(4)			0.246	

*Note.* Own calculations using data from the IHDS waves 2004 (Post=0) and 2011 (Post=1). The tables shows the TSC impact on sanitation for households with an oldest marriageable son by wealth and sex ratio The sample consists of households of all single and married males aged 15 to 34 at the time of survey. HighTSC=1 corresponds to a Grand Score for implementation of at least 61, and HighTSC=0 otherwise, as taken from WSP (2011). Districts with at least 999 women per 1000 men in the age range 15–34 (columns 1 and 2) are defined as having a ‘high sex ratio’, those with less than 999 women per 1000 men as having a ‘low sex ratio’. Ratios are computed using the 2001 and 2011 population censuses. ‘High wealth’ correspond to individuals whose asset index is above the 50th percentile of the entire country distribution per wave. Households below the cutoff are classified as having ‘low wealth’. Controls include Post, the age and marital status of the individual for whom the household is in the sample; household size; education level of the household head (no education [base], primary, incomplete secondary, secondary, and above secondary); caste (Brahmin [base], high caste, other backward caste, Dalit, Adivasi, Muslim, and Sikh or Jain); an indicator variable for being in a rural zone; and fixed effects at the district level. Standard errors clustered at the district levels are given in parentheses. A Wald test of equivalence of coefficients was performed by jointly estimating coefficients for columns 1 and 2/3 and 4 in a single regression, interacting all variables (including controls) with a dummy indicating whether the observation correspond to a ‘high wealth’ household or not. Significance: *** p < 0.01, ** p < 0.05, * p < 0.1.

### 3.2 The TSC and marital sorting on wealth

**Fig. 6:**
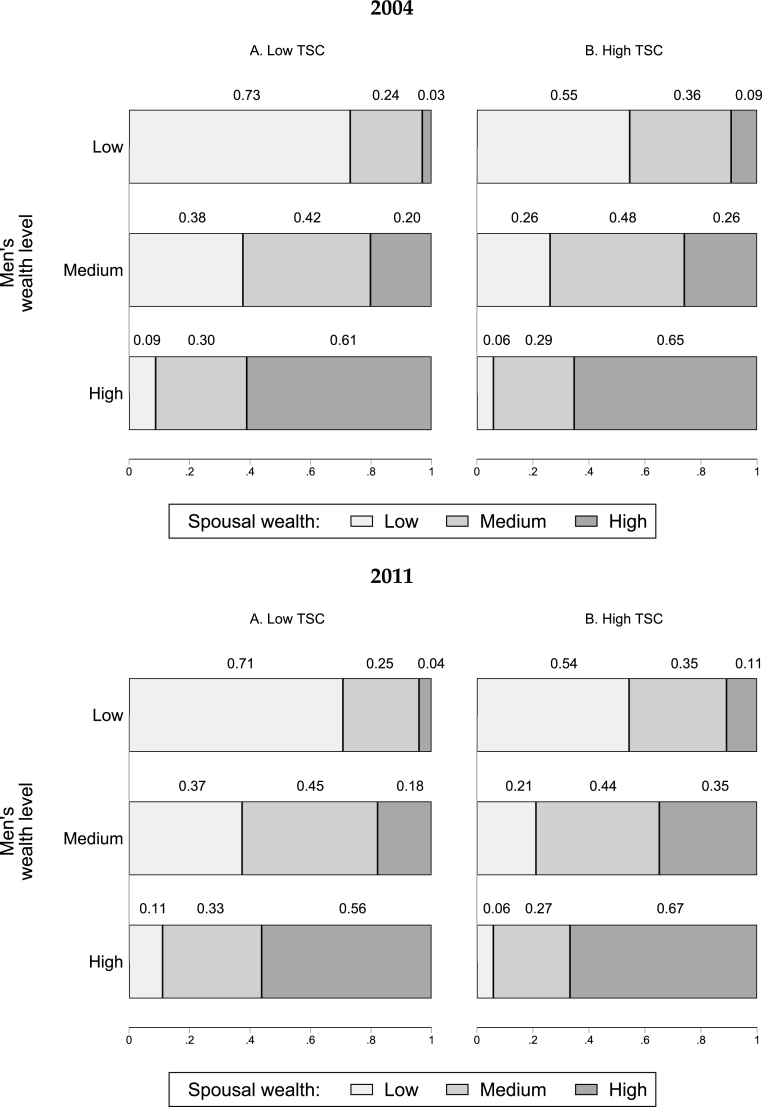
Marriage patterns of men over time across TSC exposure groups. *Note.* The sample consists of households of all single and married males aged 15 to 34 at the time of the survey. High TSC corresponds to a grand score for implementation of at least 61, and Low TSC otherwise. The Grand Score per district is taken from (WSP, 2011). ‘High wealth’ correspond to individuals whose asset index is above the 66th percentile of the entire country distribution per wave. Households below the 33th percentile cutoff are classified as having ‘low wealth’. Between the two cutoffs, households are classified as ‘medium wealth’. Proportions are computed using frequencies reported in Table A6.

In Section 2.2, we discussed the high degree of assortative matching by wealth in the Indian marriage market, which we present graphically in Fig. 6 and by high and low TSC performance for the case of men.^17^ The figure once again confirms that spouses tend to marry partners belonging to the same wealth category, and this holds in both low and high TSC districts. For example, in 2004, 73% of men with low pre-marital wealth living in low TSC areas were matched with a woman whose pre-marital wealth also fell into the same wealth category; in high TSC districts, the percentage was significantly lower at 55%. There are some differences in the matching patterns over time and across low and high TSC exposure. In 2011, the percentage of low-wealth matches in low TSC districts dropped to 71%, while in high TSC districts, it dropped from 55% to 54%.^18^ This is suggestive evidence of a potential relationship between the policy and marriage markets.

To rationalize the sorting patterns observed in the data and to examine the impact of the TSC policy on marriage market outcomes, we employ a simple matching model à la Choo and Siow (2006). The structural model helps to explain the changes in marital behavior and to quantify the policy impact on marital gains and the division of surplus, both of which are determined from the marriage market equilibrium and are thus endogenous. For instance, if TSC increases the rate of marriage for both men and women of a given wealth group, this may imply an increase in the marital surplus derived from that match. Moreover, the increased supply of sanitation, a public good within marriage, in the marriage market may generate changes in marital sorting behavior. The impact on the surplus division, however, is less straightforward and would depend on the differential policy impact by gender. In what follows, we estimate a marriage matching function, which allows for equilibrium effects (that is, substitution in spousal choice), and incorporates heterogeneity in tastes for sanitation.

## 4 Theoretical framework

In this section, we explore the impact of the TSC on the changes in matching behavior observed in Fig. 6. To do this, we explicitly model the marital choice of men and women using a simple matching model. The characterization is similar to that undertaken by Choo and Siow (2006) (henceforth, CS) and Chiappori, Salanié, and Weiss (2017) (henceforth, CSW). The structure has two interconnected elements: first, a marriage matching function that characterizes who is matched with whom and who remains single; and second, the marital surplus and surplus division that follows from the marriage decision. We begin by describing the marriage market in India using the CS transferable utility matching model.

### 4.1 Types of individuals and living arrangements

The economy comprises G separate markets, each populated by a large number of men and women who can choose to marry a member of the opposite gender or to remain single. As seen in Section 2.2, a large number of marriages take place in the same district. We, therefore, use the district as a geographic indicator for the market within which matching occurs. In what follows, we describe marriage market behavior in one district and return to the discussion of many districts in the empirical implementation.

Men and women living in a district are characterized by their wealth endowment w, which can be low, medium, or high. The set of these three wealth types is denoted by W={L,M,H}. There are thus three types of women, which are indexed by the letter J, and three types of men, which are indexed by the letter I. A matched couple is defined by their combined type IJ. The population vector of men is given by M, whose element $m_{I}>0$ denotes the measure of type I men. Similarly, the population vector of women is given by F, whose element $f_{J}>0$ denotes the measure of type J women.

In addition to individual types, a match is also characterized by the type of living arrangement K the couple chooses to live in. Living arrangement characterizes the sanitation status of the household. We distinguish between living arrangements with a toilet (K=T) and without a toilet (K=N) facility at home. The set of these two types is denoted by A={T,N}. A challenge with incorporating sanitation is that, unlike education, it is observed concurrently with the matched identity of the couple. In other words, sanitation is an attribute of the match rather than the individual.^19^ However, men and women may differ in their preference for sanitation, which may vary across the wealth distribution. Given that individuals simultaneously choose their preferred spouse and living arrangement, the variation in the preference for sanitation across gender and wealth, in turn, affects the matching behavior and marital prospects.

Each male and female type is associated with a utility function, a value of pre-marital wealth, and a distribution of preference shocks. Although we impose some restrictions in our empirical analysis, in principle, all these objects can vary across districts. In the benchmark model, initial wealth fully defines the match-relevant type space for men and women. The implicit assumption is that men and women differ only in their wealth endowment. However, an individual’s type may be characterized by a vector of personal and socioeconomic attributes. To allow for differences across individuals, we introduce heterogeneity in the marital preferences below. Doing so allows for the fact that a man I’s (woman J’s) preference to match with a woman J (man I) may depend on his (her) own characteristics, such as age and education.

In total, there are 18 (W×W×A) different types of marriages, as given by all possible combinations of husband and wife types IJ and living arrangements A={T,N}. A marriage matching function is a W×W×A matrix μ whose (I,J,K) element $\mu_{IJK}$ describes the measure of type I men married to type J women in living arrangement K. To allow for the possibility that some individuals choose not to marry, unmatched men and women are denoted as choosing partner type 0 in living arrangement 0. In this case, $\mu_{I00}$ and $\mu_{0J0}$ denote measures of single men of type I and single women of type J, respectively. Lastly, we use the notation i (j) to indicate an individual male (female) of a given type I (J), respectively.

### 4.2 Gains from marriage

***Marital output.*** If married, a man of type I and a woman of type J generate together a marital output that they can divide between them. In our model, the primary purpose of marriage is the consumption of household public goods. The output is characterized by a systematic component ($\zeta^{IJK}$) and an idiosyncratic component ($\theta^{IJK}$). We assume that the systematic component depends on the wealth of both partners as well as the living arrangement and is given by

(2) $$\zeta^{IJK}=\underset{I}{\sum }\underset{J}{\sum }\alpha^{IJ}w^{IJ}+\underset{I}{\sum }\underset{J}{\sum }\underset{K}{\sum }\delta^{IJK}a^{IJK}$$

where $w^{IJ}$ is a dummy variable that takes on a different value for each of the nine possible combinations for husband and wife wealth, while $a^{IJK}$ is a dummy variable that takes on a different value for each of the eighteen possible combinations for couple wealth and living arrangement. Note that the living arrangement enters in an additively separable way, which allows us to rank marriages by household sanitation status. The parameter $\alpha^{IJ}$ accounts for the strength of mutual attractiveness across the wealth of men and women and does not vary by living arrangement. In contrast, the parameter $\delta^{IJK}$ captures the importance of the two living arrangements (T, N), which varies with the wealth of the couple. For instance, $\delta^{LLT}\neq \delta^{HHT}$ may reflect an underlying difference in the desire for sanitation by wealth. This parameterization allows for the taste for sanitation, a household public good, to vary with the joint wealth of the household as well as across gender. The marital output in Eq. (2) denotes the maximal attainable utility for the household and is determined by the allocation decision of the joint household wealth towards public and private consumption. It reflects the preferences of the individuals and depends on the price of sanitation and joint household wealth. More specifically, the marital output allows us to characterize the benefits that accrue from the joint consumption of sanitation. These benefits might include cost-sharing gains as well as direct benefits that may be gender specific.

In a given marriage (I,J,K), the marital output $\zeta^{IJK}$ is constant. The assumption of transferable utility implies that upon dividing the marital output, utility is transferred between the husband and the wife at a constant exchange rate. In addition to the systematic component $\zeta^{IJK}$, partners receive a utility from the quality of their match $\theta_{ij}^{IJK}$ that is unobservable to the researcher. Partners may have different valuations of the marriage, with θ denoting the sum of these valuations. Thus, the total marital output generated by a match is $\zeta^{IJK}+\theta_{ij}^{IJK}$.

***Singlehood.*** Single men and women receive utility based on their type. Like the marital output, the utility for singles includes a systematic and an idiosyncratic component, which represent the individual preferences to remain single. The utility of being single for a man i of type I is given by

(3) $$U_{i}^{I00}={\overset{\sim }{u}}^{I00}+{ɛ}_{i}^{I00}$$

where the systematic utility ${\overset{\sim }{u}}^{I00}$ is common to all type I men who remain single. Similarly, the utility of being single for a woman j of type J is given by

(4) $$V_{j}^{0J0}={\overset{\sim }{v}}^{0J0}+\eta_{j}^{0J0}$$

where the systematic utility ${\overset{\sim }{v}}^{0J0}$ is common to all type J women who remain single. The idiosyncratic taste component is denoted by shocks ${ɛ}_{i}^{I00}$ for men and $\eta_{j}^{0J0}$ for women. The taste shocks are specific to an individual i (j) and are assumed to be independent and identically distributed random variables. Because we are dealing with a discrete choice model, some normalizations are required. Given the static structure, the systematic utility of being single is normalized to zero for both men and women.

***Marital surplus.*** Using this characterization of the economic environment, we can introduce the equation for the total marital surplus generated by the match between man i of type I and woman j of type J living in arrangement K:

(5) $$\pi_{ijK}=\underset{\pi^{IJK}}{\underset{︸}{\zeta^{IJK}-{\overset{\sim }{u}}^{I00}-{\overset{\sim }{v}}^{0J0}}}+\left({ɛ}_{i}^{IJK}-{ɛ}_{i}^{I00}\right)+\left(\eta_{j}^{IJK}-\eta_{j}^{0J0}\right)$$

The total marital surplus characterizes the returns from the marriage IJK (i.e., the gains from being married relative to being single), which includes a systematic and idiosyncratic component. The systematic component $\pi^{IJK}$ denotes the joint marital surplus that depends only on the marriage type. As $\pi^{IJK}$ is a function of the marital output, we can relate changes in the marital surplus to changes in the price of sanitation induced by the TSC policy described in Section 2. In addition, ɛ and η each denote a vector of gender-specific idiosyncratic preference shocks representing taste heterogeneity. The preference shocks often characterized as love shocks can be interpreted, in a parsimonious way, as the “quality” of the match $\theta_{ij}^{IJK}$ (Chiappori, 2020), where $\theta_{ij}^{IJK}={ɛ}_{i}^{IJK}+\eta_{j}^{IJK}$. The preference heterogeneity components are assumed to be additively separable in the surplus function and depend only on the partner’s type I (or J), not his (or her) exact identity i (or j).

### 4.3 Marital preference over types

The joint marital surplus in Eq. (5) can be shown to be an outcome of a set of discrete choice problems for each man and woman participating in the marriage market choosing whether and whom to marry. This decentralization relies on the additive separability of the taste shocks ɛ and η in Eq. (5). Let the utility of man i of type I who matches with a woman of wealth type J in an arrangement K be

(6) $$U_{i}^{IJK}={\overset{\sim }{u}}^{IJK}-\tau^{IJK}+{ɛ}_{i}^{IJK}$$

where ${\overset{\sim }{u}}^{IJK}$ denotes the systematic gross utility common to all males of type I matching with a female of type J in an arrangement K, while $\tau^{IJK}$ is the equilibrium transfer. In order for man I to form a match, he must transfer to his prospective spouse a part of his utility that he values at $\tau^{IJK}$. The idiosyncratic component of male marital preference ${ɛ}_{i}^{IJK}$ measures the departure of each individual male i’s utility $U_{i}^{IJK}$ from the systematic component, which is common to all men I who marry women J. These shocks allow observationally identical individuals to make different choices with regard to remaining single or marrying a particular type of spouse in a specific living arrangement. The utility of type I man i who remains unmatched is given by Eq. (3).

The marriage decision problem for a man i of type I is to choose to marry one of the W={L,M,H} types of women or to remain single. A male i of type I will choose according to

(7) $$U_{i}^{I}=\max\;\left\{U_{i}^{I00},U_{i}^{ILT},U_{i}^{IMT},U_{i}^{IHT},U_{i}^{ILN},U_{i}^{IMN},U_{i}^{IHN}\right\}$$

As mentioned previously, we assume the idiosyncratic taste terms are independent and identically distributed according to the type 1 extreme value distribution.^20^ This assumption implies that the proportion of type I men who would like to marry a type J woman under arrangement K or remain unmarried is given by the conditional choice probabilities:

(8) $$Pr\left(J\;and\;K|I\right)=\frac{{\left(\mu_{IJK}\right)}^{d}}{m_{I}}=Pr\left[U_{i}^{IJK}>\max\;\left\{U_{i}^{IJ^{\prime }K^{\prime }},U_{i}^{I00}\right\}\;\forall \;J^{\prime }\neq J,\;K^{\prime }\neq K\right]=\frac{exp\left({\overset{\sim }{u}}^{IJK}-\tau^{IJK}\right)}{exp\left({\overset{\sim }{u}}^{I00}\right)+\underset{K^{\prime }\in \left\{T,N\right\}}{\sum }\overset{H}{\underset{J=L}{\sum }}exp\left({\overset{\sim }{u}}^{IJK^{\prime }}-\tau^{IJK^{\prime }}\right)}$$

where $m_{I}$ denotes the number of men of type I and ${\left(\mu_{IJK}\right)}^{d}$ is the number of (I,J,K) matches demanded by type I men. Using Eq. (8), we can derive the familiar log odds ratio as follows:

(9) $$\ln\frac{{\left(\mu_{IJK}\right)}^{d}}{{\left(\mu_{I00}\right)}^{d}}={\overset{\sim }{u}}^{IJK}-{\overset{\sim }{u}}^{I00}-\tau^{IJK}$$

where ${\left(\mu_{I00}\right)}^{d}$ is the number of type I men who remain single. Eq. (9) is the quasi-demand equation for type I males demanding (I,J,K) matches.

The choice problem for women can be defined in a similar manner to that of men. The random utility function used for women is similar to that used for men except that in the matching with a type I man in an (I,J,K) match, a type J woman receives the transfer $\tau^{IJK}$, while the utility of type J woman j who remains unmatched is given by Eq. (4). The idiosyncratic marital preference of type J woman j is also assumed to be independently and identically distributed with a type 1 extreme value distribution. In this case, the quasi-supply equation of type J females for (I,J,K) matches is given by

(10) $$\ln\frac{{\left(\mu_{IJK}\right)}^{s}}{{\left(\mu_{0J0}\right)}^{s}}={\overset{\sim }{v}}^{IJK}-{\overset{\sim }{v}}^{0J0}+\tau^{IJK}$$

where ${\left(\mu_{IJK}\right)}^{s}$ is the number of (I,J,M) matches offered by type J women and ${\left(\mu_{0J0}\right)}^{s}$ is the number of type J women who want to remain unmatched. Eq. (10) is the quasi-supply equation for type J females for (I,J,K) matches.

***Division of surplus.*** Note that in equilibrium, Eq. (9), (10) are directly related to the division of the surplus between men and women. As the shocks to the preferences of individuals are related to the types of spouses whom they marry and not to individuals, men and women available on the market are indifferent between marrying different individuals of the same type. As a consequence, in equilibrium, these individuals must receive the same share of the expected marital surplus in a given type of marriage. Let $\gamma^{IJK}$ be the share of the systematic marital surplus that is obtained by a man of type I in arrangement K who marries a woman of type J where the woman receives $\left(1-\gamma^{IJK}\right)$. The parameters $\gamma^{IJK}$ and $\left(1-\gamma^{IJK}\right)$ reflect the share from the systematic surplus of the marriage that men and women expect to receive at the time of marriage.

### 4.4 Marriage market equilibrium

The 18 (W×W×A) marriage sub-markets clear when, given equilibrium transfers $\tau^{IJK}$, the demand by type I men for (I,J,K) relationships is equal to the supply of type J women for (I,J,K) relationships for all (I,J,K):

(11) $${\left(\mu^{IJK}\right)}^{d}={\left(\mu^{IJK}\right)}^{s}=\mu^{IJK}$$

Substituting Eq. (11) into Eqs. (9), (10) and adding the two equations yields the following:

(12) $$\ln\frac{\mu_{IJK}}{\mu_{I00}}+\ln\frac{\mu_{IJK}}{\mu_{0J0}}=\underset{\zeta^{IJK}}{\underset{︸}{{\overset{\sim }{u}}^{IJK}+{\overset{\sim }{v}}^{IJK}}}-{\overset{\sim }{u}}^{I00}-{\overset{\sim }{v}}^{0J0}$$

Eq. (12) highlights two important points. First, the right-hand side denotes the joint marital surplus where ${\overset{\sim }{u}}^{IJK}$ and ${\overset{\sim }{v}}^{IJK}$ denote the shares of the marital output. The left-hand side denotes the division of the surplus between men and women, which can be defined as $\gamma^{IJK}\pi^{IJK}$ and $\left(1-\gamma^{IJK}\right)\pi^{IJK}$, respectively. Second, the marriage matching function can be derived by rewriting Eq. (12) as

(13) $$\pi^{IJK}=\ln\frac{{\left(\mu_{IJK}\right)}^{2}}{\mu_{I00}\cdot \mu_{0J0}}$$

Eq. (13) has an intuitive interpretation where the left-hand side denotes the total marital surplus for a match (I,J,K) while the right-hand side denotes a log transformation of the marriage matching function characterized by the ratio of the number of (I,J,K) marriages to a geometric average of the number of singles. The marital surplus reflects the total marital gain for any couple (I,J) matched in living arrangement K *relative* to the total gain from remaining unmarried. In other words, the difference $\left(\pi^{IJT}-\pi^{IJN}\right)$ reflects the attractiveness of matches with a toilet to matches without a toilet relative to singlehood for a given match (I,J).

Lastly, the marriage matching function is a W×W×A matrix whose (I,J,K)th element is $\mu_{IJK}$. The function must satisfy the following accounting constraints:

(14) $$\mu_{I00}+\underset{K\in \left\{T,N\right\}}{\sum }\underset{J\in \left\{L,M,H\right\}}{\sum }\mu_{IJK}=m_{I}\;\;\forall \;\;I\;\in \;W$$

(15) $$\mu_{0J0}+\underset{K\in \left\{T,N\right\}}{\sum }\underset{I\in \left\{L,M,H\right\}}{\sum }\mu_{IJK}=f_{J}\;\;\forall \;\;J\;\in \;W$$

(16) $$\mu_{I00},\;\mu_{0J0},\;\mu_{IJK}\;\geq 0\;\;\forall \;\;\left(I,J,K\right)\;\in \;W\times W\times A$$

where the first equation denotes that the number of available type I men $m_{I}$ must be equal to the total number of men I who marry type J women under both living arrangements plus the number of single men. The constraint must hold for all male types I∈W={L,M,H}. The second equation, meanwhile, provides a similar set of accounting conditions that must hold for all female types J. The last accounting constraint holds because the number of unmarried persons of any type and gender and the number of marriages (I,J,K) must be non-negative. These accounting constraints are crucial to ensuring that the predicted marriage rates do not exceed 1, i.e., the matching is feasible.

### 4.5 Empirical implementation

The model is estimated using data from the IHDS. In terms of marital behavior, for each type, we observe the proportion of single men, the proportion of each type of single woman, and the quantity of type I men married to J women ($\mu_{IJK}$) under each living arrangement (W×W×A observations). Following the characterization of the marriage decisions of men in Eq. (8), we also observe the proportion of matches between type I men and type J women in each living arrangement K conditional on being a type I male. A similar conditional probability is observed for women of each type J.

#### 4.5.1 Identification

To apply the model to the data, we implement the type 1 extreme value assumption for idiosyncratic marital tastes mentioned previously.

***Division of surplus.*** To identify the division of the surplus, we make use of the conditional choice probabilities characterizing the male and female marriage decisions. As seen from Eqs. (9) and (10), the extreme value structure of the preference shocks to the individuals implies that the observed ratio of choice probabilities identifies the difference in utility between any two marital choices. For example, the difference in utility for a type I man between marrying a wife of type J and living in arrangement K or remaining single can be expressed as

$${\overset{\sim }{u}}^{IJK}-{\overset{\sim }{u}}^{I00}-\tau^{IJK}\;=\ln\left(\frac{Pr\left(\text{man of type}I\text{selects wife of type}J\text{and arrangement}K\right)}{Pr\left(\text{man of type}I\text{is single}\right)}\right)$$

Similarly, the difference in utility for a type J woman between marrying a man of type I and living in arrangement K or remaining single can be expressed as

$${\overset{\sim }{v}}^{IJK}-{\overset{\sim }{v}}^{0J0}+\tau^{IJK}\;=\ln\left(\frac{Pr\left(\text{woman of type}J\text{selects husband of type}I\text{and arrangement}K\right)}{Pr\left(\text{woman of type}J\text{is single}\right)}\right)$$

The expressions above imply that the marital choices of available men identify the proportion of the joint marital surplus that men receive in different marriage types, where $\gamma^{IJK}\pi^{IJK}$ denotes the male share in marriage IJK. In an analogous way, the marital choices of available women identify the amount of the joint marital surplus that women receive in different marriage types, where $\left(1-\gamma^{IJK}\right)\pi^{IJK}$ denotes the female share in marriage IJK.

Moreover, the ratio of the choice probabilities for different types of individuals can be combined to identify the marital surplus share of husbands and wives in each type of marriage:

(17) $$\frac{\left(1-\gamma^{IJK}\right)}{\gamma^{IJK}}=\frac{\ln\left(\frac{Pr\left(\text{woman of type}J\text{selects husband of type}I\text{and arrangement}K\right)}{Pr\left(\text{woman of type}J\text{is single}\right)}\right)}{\ln\left(\frac{Pr\left(\text{man of type}I\text{selects wife of type}J\text{and arrangement}K\right)}{Pr\left(\text{man of type}I\text{is single}\right)}\right)}$$

In other words, the marital surplus shares are identified from the willingness of men of type I to enter into an IJK marriage relative to the willingness of women of type J to enter into that same marriage. Lastly, as evident from Eq. (12), data on the proportions of people who get married identify both the marital surplus received jointly by men and women and the marital surplus share $\gamma^{IJK}$. As a result, our benchmark model is left under-identified.

To proceed further, we make two adjustments. First, we normalize the utility from singlehood to zero for men and women. In this case, all estimated parameters of the marriage utility are interpreted relative to the normalization. The normalization still leaves a just-identified model where the number of equations in Eq. (12) is equal to the number of unknown parameters. Second, we make use of matching data from the 345 districts observed in our sample to increase the number of equations relative to the number of unknowns in order to identify the model primitives and generate over-identifying restrictions.

***Multiple markets.*** The crucial feature of our identification approach, like others in the literature, is that we observe and thereby are able to exploit multiple observations of the marriage market. In practice, we consider several districts indexed by g=1,…,G and exploit across-district variation in the TSC exposure of the populations. Observing behavior across several markets generates the identifying restrictions for the model. This can be explained with the help of Eq. (18) below, which illustrates the identification problem as well as the intuition behind the restrictions that aid in identification. More specifically, to identify the surplus matrix $\pi^{IJK}$ and generate testable restrictions, we include gender-specific drifts:

(18) $$\pi_{g}^{IJK}=\pi^{IJK}+\nu_{g}^{I}+\xi_{g}^{J}$$

Eq. (18) allows the surplus term to vary across districts in a specific way, whereby the drift parameters capture potential differences across districts in the surplus generated by marriage.

The use of “multiple markets” is widespread in the empirical literature on marriage markets. There are two advantages to using a market definition that exploits cross-sectional variation in observed behavior that is specific to our analysis. First, using cross-district variation requires assumptions on the independence of observed matching behavior across districts. The validity of such assumptions relies on the limited inter-district marriage interactions observed in our data, as discussed in Section 2.2. Moreover, cross-sectional market variation does not impose strong restrictions on time invariance of the marital surplus within a district over time. Several papers have used the multi-market idea by using time variation in male and female characteristics in the population, which requires assumptions on the independence across different age cohorts; see, for example, CSW. Second, given that the TSC policy exposure variable operates at the district level, we can use a simple difference-in-difference type estimator like Eq. (1) in Section 3.1 to decompose the impact of the TSC on the marital surplus estimates at the district level.^21^

#### 4.5.2 Estimation

We use a minimum distance framework adopted from CSW to estimate the marital surplus in Eq. (12), which can be rewritten as

(19) $$\ln\left(Pr\left(J\;and\;K|I,g\right)Pr\left(I\;and\;K|J,g\right)\right)=\pi_{g}^{IJK}\equiv G_{IJK}+E_{g}^{I}+F_{g}^{J}$$

where the unknown object $\pi_{g}^{IJK}$ is constrained by a set of parameters $G_{IJK}$ and $E_{g}^{I}$ and $F_{g}^{I}$, which are the parameters of interest. Following the parameterization of the marital surplus in Eq. (19), the parameter $G_{IJK}$ captures the cross-district invariant component of the marital surplus, while $E_{g}^{I}$ and $F_{g}^{J}$ are gender-specific parameters that account for fixed differences in the marital surplus across districts. For instance, the estimates of $E_{g}^{I}$ and $F_{g}^{I}$ capture cultural differences in marriage practices that may be region- and gender-specific and contribute to changes in the marital surplus across districts.

Although $\pi_{g}^{IJK}$ is an unknown, Eq. (19) shows us that there is a mapping between the behavior observed in the data and the structural primitives of the model. In other words, the model is estimated in the same way that it is identified using Eq. (19), which also represents a statistical model that can be taken to the data. The sampling variation arises from the cross-district variation in the probability estimates on the left-hand side of the equation. To proceed, we obtain an estimate of the deterministic part of the joint surplus $\pi_{g}^{IJK}$ by combining estimates of the conditional matching probabilities:

(20) π^gIJK=lnP^(JandK|I,g)P^(IandK|J,g)

The estimated probabilities are simple averages that are observed in the data. Within each district g, we observe the proportion of men and women of different types along with the proportion of marriages with and without sanitation. The estimator of the probability that man i of type I matches with a woman of wealth J and in a living arrangement with sanitation (K=T) is simply the proportion of that group of men married to a type J woman in marriages with sanitation. In practice, however, we face a thin cell problem in constructing these averages. We have a small number of observations per district, which leads to many empty cells for certain match pairings. To overcome this challenge, we use a two-step conditional choice probability (CCP) estimator where, in the first stage, the choice probabilities on the left-hand side of Eq. (20) are computed as predicted probability estimates from a parametric regression specification. When constructing the CCP estimate, we take into account the sampling weights provided in the IHDS data.

In the second stage, we use a minimum distance framework adopted from CSW to estimate the marital surplus in Eq. (19) where we substitute $\pi_{g}^{IJK}$ with its empirical counterpart π^gIJK constructed in the first stage. The minimum distance estimator proposed by CSW can be used to obtain estimates of the parameters $G_{IJK}$, $E_{g}^{I}$ and $F_{g}^{J}$ by choosing $\left(G_{IJK},E_{g}^{I},F_{g}^{J}\right)$ to minimize

(21) $$\overset{G}{\underset{g=1}{\sum }}\epsilon_{g}^{\prime }W_{g}\epsilon_{g}$$

where $\epsilon_{g}={\overset{ˆ}{\pi }}_{g}^{IJK}-G_{IJK}-E_{g}^{I}-F_{g}^{I}$ and $W_{g}$ is a matrix that converges to the inverse of the variance–covariance matrix of the vector $\overset{ˆ}{\pi }$ that stacks all the estimates ${\overset{ˆ}{\pi }}_{g}^{IJK}$. Note that the parameters are estimated by minimizing the expression in Eq. (21) where $\epsilon_{g}$ is *linear* in the unknown parameters. For the framework used by CS, Decker et al. (2013) show that the solution is locally unique.

**Fig. 7:**
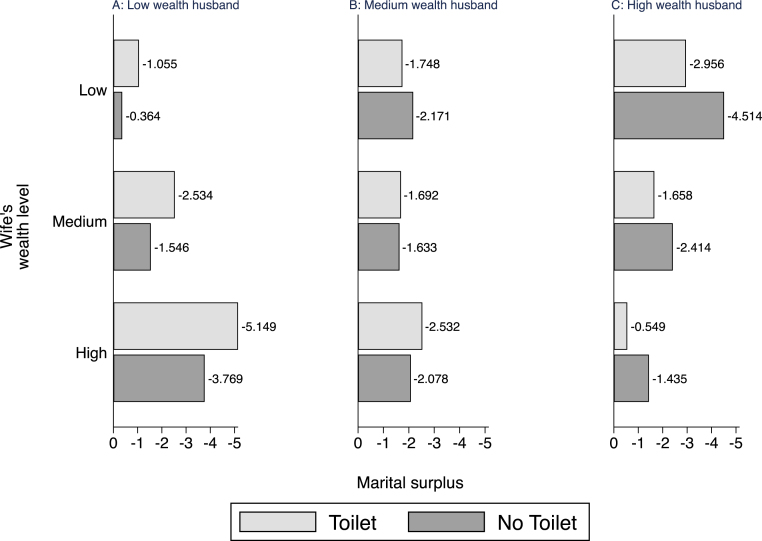
Marital Surplus. *Note.* The graph presents the marital surplus estimates for a given couple IJ in marriages with toilet ${\overset{ˆ}{\pi }}^{IJT}$ and without toilet ${\overset{ˆ}{\pi }}^{IJN}$. The three panels correspond to the wealth of the husband, while the columns correspond to the wealth of the wife. All differences between the living arrangements are significant at the 95% level. Exact numbers are available in Appendix Table A7.

#### 4.5.3 Model estimates

We now discuss the estimated marital surplus and the estimated value of sanitation in marriage. In Section 5, we present and discuss the TSC policy impact on the marital surplus and the surplus shares received by husbands and wives. To demonstrate the difference in marital gains between marriages with and without sanitation, we show in Fig. 7 the systematic total gains in marriages for different living arrangements, i.e., with ($\pi^{IJT}$) and without ($\pi^{IJN}$) sanitation for couples of the same wealth type in 2004 prior to TSC exposure. Recall that the marital gain measures the expected marital surplus to a random (I,J,K) pair marrying relative to them not marrying.

Fig. 7 highlights two key points. First, the marital surpluses under both living arrangements lie below zero, indicating the large fraction of younger women and men in our sample. We discuss this further in the next section. Second, the gains to marriage with sanitation are not unilaterally larger but instead vary with the wealth distribution across the marriage sub-markets, thus highlighting the scope for large heterogeneous policy effects. Specifically, the marital gains in marriages with sanitation are higher than those in marriages without where the man has high wealth and where the man has median wealth and the woman low.

## 5 The TSC impact on marriage markets

From 2004 to 2011, the TSC policy underwent its final expansion phase, which provided subsidies to households to build sanitation facilities at home. This decline in the price of household public goods gave households a greater incentive to adopt sanitation, and many of these households had individuals active in the marriage market. In this section, we explore what, if any, impact the TSC had on marriage outcomes, such as the marital surplus and the female (or male) surplus share.

### 5.1 Impact on marital surplus

To evaluate the overall impact of the TSC, we first consider the impact on the aggregate district-level marital surplus. The policy exposure variable divides districts into two categories: those with high and those with low exposure to the TSC during this period. If high TSC exposure in a district increased gains to marriage within that district, we would expect to see larger gains to marriage in the high TSC districts than in the low TSC districts in the post-policy period.^22^

The top panel of Table 3 presents the marital surplus estimates for spouses of different wealth types and matches, averaged over the two possible types of living arrangement, with and without sanitation. All of the marital surpluses are negative. This absolute level of utility is arbitrary in our model and follows from the normalization of utility from remaining single. More specifically, a single person can choose whether to remain single or marry into one of 18 different types of marriages. In our data, the proportion of men and women who enter into a particular type of marriage is smaller than the proportion remaining single. It thus follows from the model that the utility of entering a particular marriage must be smaller than the utility of remaining single. This implies that the expected marital surplus must be negative. The meaningful content in the top panel of Table 3, therefore, is the difference between the marital surplus in different types of marriage. In each row, we see that the marital surplus weakly decreases in matches off the main diagonal. This is because most matches occur among couples with the same wealth. The larger marital surpluses along the diagonal reflect the relative attractiveness of matches among couples of similar wealth.

**Table 3:** Average match surplus ${\overset{ˆ}{\pi }}^{IJ}$.

**Wife**→	Wealth type L	Wealth type M	Wealth type H
**Husband**↓	${\overset{ˆ}{\pi }}^{IJ}$		
Wealth type L	−0.988	−2.412	−5.067
	(0.026)	(0.051)	(0.154)
Wealth type M	−2.705	−2.404	−3.246
	(0.033)	(0.023)	(0.049)
Wealth type H	−4.307	−2.705	−1.568
	(0.067)	(0.026)	(0.030)

**Husband**↓	TSC impact on ${\overset{ˆ}{\pi }}^{IJ}$		

Wealth type L	0.148	−0.623***	−0.576*
	(0.105)	(0.126)	(0.346)
Wealth type M	0.123	−0.429***	0.152
	(0.118)	(0.051)	(0.101)
Wealth type H	0.401*	0.234**	0.061
	(0.238)	(0.077)	(0.061)

*Note.* The top panel presents the weighted average marital surplus estimates across both living arrangements denoted by ${\overset{ˆ}{\pi }}^{IJ}$. The estimates are constructed using a weighted average of all estimated marital surpluses occurring in different marriage types; weights are given by the proportion of living arrangements with and without sanitation within a match type IJ. The bottom panel presents the TSC policy impact on marital surplus, where each cell presents the difference-in-difference estimate. Wealth types L, M and H refer to low, medium and high wealth, respectively. Standard errors, shown in parentheses, are clustered at the district level and computed using 1000 bootstrap replications. Significance: *** p < 0.01, ** p < 0.05, * p < 0.1.

We next discuss the impact of the TSC policy on the marital match surplus, which – as we show in Table A8 – is increasing in aggregate. This overall increase in marital surplus is explained by an increase in the number of marriages relative to singlehood. In other words, marriage becomes a more attractive alternative to singlehood as a result of TSC. Although the aggregate marital surplus increased more in high TSC than in low TSC districts, the accrual of these gains was not homogeneous across the match types, as shown in the lower panel of Table 3. The results showing the impact of the TSC policy on the marital surplus for a couple in each combination of wealth types illustrate two main findings. First, the increase in marital surplus is not uniform across the match types. Although not all significant, the increases are mostly realized in the lower triangle of the matrix in Table 3. In other words, gains are largely restricted to households with wealthy men. In particular, high TSC exposure leads to a statistically significant increase in match surplus for two couple types, namely, where high-wealth men are matched with women of low or medium pre-marital wealth. At the same time, we find that three out of the nine match types experience a statistically significant *decrease* in their match surplus due to the TSC policy. These are couples where the man has low pre-marital wealth and marries a woman of higher wealth status (medium or high), as well as men with medium pre-marital wealth who marry women of the same type.

Second, the distribution of the gains and losses is not symmetric. As seen in the lower panel of Table 3, increases in marital surplus occur in matches where the husband is as wealthy as, if not wealthier than, the wife, while decreases in marital surplus occur in matches where the wife is wealthier than the husband. Comparing the two extreme entries on the anti-diagonal illustrates this point clearly. Matches between low-wealth males and high-wealth females experience a significant decrease in marital surplus. These marriages were not an attractive option prior to the policy introduction, as seen from the top panel, and are significantly less so after policy exposure. This decline stands in stark contrast to the significant increase in marital surplus for matches between a high-wealth male and a low-wealth female. Overall, the findings in Table 3 suggest that not only did the TSC affect the aggregate marital surplus, but it may have also shifted the gains in an unequal way, favoring matches where the husband was wealthier than the wife.

***Impact on sorting.*** We use the estimated model to analyze how the TSC affected outcomes in marriage markets. The marriage rate for men (women) of type I (J) is given by $\left(1-\frac{\mu^{I00}}{m_{I}}\right)$ $\left(1-\frac{\mu^{0J0}}{f_{J}}\right)$. Table 4 shows how the marriage rate of men and women was affected by the TSC. The estimates capture how high exposure to TSC changed the overall gains from entering the marriage market and reflect the choice of *whether* to marry. We find that in equilibrium both poor men and poor women are more likely to marry with TSC exposure. In contrast, we do not find a statistically significant increase among individuals of medium and high wealth. The impacts in Table 4 are in line with our previous estimates in Table 3 on marital surplus, which suggests that the increase in gains from marriage in districts with high TSC exposure is accompanied by an increase in the sorting of men and women into marriage.

**Table 4:** TSC impact on marriage rate .

	Marriage rate	
	Men	Women
	(1)	(2)
Wealth type L	0.037***	0.073***
	(0.006)	(0.008)
Wealth type M	0.008	0.001
	(0.005)	(0.006)
Wealth type H	−0.002	−0.003
	(0.004)	(0.007)

*Note.* This table presents the TSC policy impact on the marriage rate for men (column 1) and women (column 2). Wealth Types L, M and H refer to low, medium and high wealth. Each cell presents the difference-in-difference estimate. The base marriage rate among men: 0.435 (type L), 0.392 (type M), and 0.347 (type H). The base marriage rate among women: 0.653 (type L), 0.523 (type M), and 0.351 (type H). Regression specification includes district and time fixed effects. Household level controls include the age of the man (or woman) and caste. Standard errors, shown in parentheses, are clustered at the district level and computed using 1000 bootstrap replications. Significance: *** p < 0.01, ** p < 0.05, * p < 0.1.

Furthermore, we see that the greatest policy impact is concentrated among individuals at the lower end of the wealth distribution.^23^ This is because the introduction of the TSC policy implied an effective decrease in the price of sanitation, a household public good, within the exposed district. As a result, households (mostly poor) in high TSC districts are more able to build a sanitation facility at home than their counterparts in low TSC districts. Table 4 suggests that the increase in sanitation was accompanied by an increase in the marriage rate of poor men and poor women who would otherwise not have married at that given point in time. A large decrease in the price of a household public good induces people to marry. Although the price of sanitation decreases for all households – married and single alike – it is notable that within married households, individuals enjoy additional cost-sharing gains from the joint consumption of the public good relative to a single household. In other words, within a couple, each individual’s contribution to the public good is necessarily lower than in a single household without joint consumption. In this scenario, a decrease in the price of public good makes marriage a far more attractive alternative relative to singlehood. Note that the significant difference in impact between men and women is simply an outcome of the different distribution of men and women in each wealth category. Lastly, Tables A11 and A12 in the appendix show how the partner choice of men and women changes in response to the TSC policy. These probabilities denote the spousal choice of men and women conditional on being married. Note that the discrepancy between the two choice probabilities arises as a result of having a different distribution of men and women in each wealth category.

### 5.2 Impact on surplus share

A question that arises from the finding of increased marital surplus, as shown in Table 3, is how the additional marital utility derived from TSC exposure is distributed within the marriage. The distribution here refers to the allocation of residual resources, i.e., after the public good spending has been decided. Using the estimated parameters for the male and female gains from marriage averaged over different living arrangements, Table 5 illustrates the overall impact of the TSC on the female surplus share. The table set-up is analogous to Table 3 in that the upper panel shows the female surplus shares for the nine different match types averaged over the two possible types of living arrangement, while the lower panel shows the impact that living in a high TSC district has on these shares. We find that the majority of coefficients are negative (7 out of 9), three statistically significantly so. Specifically, women of low pre-marital wealth who marry a man of higher wealth status (M or H), as well as women of high pre-marital wealth who marry a partner from the same wealth category, experience a statistically significant decrease in their surplus share after being exposed to the TSC policy.

**Table 5:** Female surplus share $\left(1-\gamma^{IJ}\right)$.

**Wife**→	Wealth type L	Wealth type M	Wealth type H
**Husband**↓	$\left(1-\gamma^{IJ}\right)$		
Wealth type L	0.542	0.531	0.531
	(0.007)	(0.012)	(0.031)
Wealth type M	0.493	0.441	0.426
	(0.018)	(0.009)	(0.022)
Wealth type H	0.549	0.645	0.457
	(0.034)	(0.014)	(0.007)

**Husband**↓	TSC impact on $\left(1-\gamma^{IJ}\right)$		

Wealth type L	−0.007	−0.039	−0.450**
	(0.026)	(0.071)	(0.183)
Wealth type M	−0.453***	0.099	−0.087
	(0.138)	(0.065)	(0.133)
Wealth type H	−0.187**	−0.006	0.051
	(0.109)	(0.097)	(0.031)

*Note.* The top panel presents the weighted average of female surplus share estimates across both living arrangements denoted by $\left(1-\gamma^{IJ}\right)$. The bottom panel presents the TSC policy impact on female surplus share, where each cell presents the difference-in-difference estimate. Wealth Types L, M and H refer to low, medium and high wealth. Standard errors, shown in parentheses, are clustered at the district level and computed using 1000 bootstrap replications. Significance: *** p < 0.01, ** p < 0.05, * p < 0.1.

Table 5 highlights two key takeaways. First, the TSC impact on surplus share indicates a gender-specific response to the price subsidy. This suggests that men and women value the public good differently and that this difference in valuation could be reflected in the allocation of residual household resources. Second, the decline in female surplus share can be understood as a corresponding decrease in women’s control over residual household resources. This decrease can be rationalized by a smaller female share of private goods in exchange for a different marriage arrangement in response to the price subsidy. Although the IHDS does not provide information on individual-level consumption, we provide corroborative evidence for the decline of female surplus share using several measures of women’s control over household resources in Appendix C.^24^ It is important to note that our results do not imply that the woman is necessarily worse off ex-post TSC exposure. The total marital utility is higher, and so in equilibrium, we see more marriages and, thus, the entry of men and women.

Table 5 also illustrates the importance of equilibrium effects where the underlying sharing weight is not a fixed parameter solely dependent on the scarcity of women relative to men. In addition to matching attributes, the surplus share of husbands and wives is also determined by their willingness to enter the marriage market. In other words, whereas for some types of couples, there are benefits to getting married as seen in Table 3, wives will receive a smaller share of the surplus. This implies that the intra-household distribution of resources is not a policy invariant parameter but an endogenous entity reflecting marriage market conditions (Chiappori et al., 2018).

## 6 Conclusion

While the case for investment in sanitation has generally been convincingly made, there remains an incomplete understanding of the impacts of sanitation interventions (Augsburg and Sainati, 2020, Gautam, 2017). This may be due to the complexity and heterogeneity of sanitation, which implies direct monetary and non-monetary costs and benefits, many of which are difficult to measure, as well as the presence of externalities (Gautam, 2023). Moreover, there may be indirect impacts on individuals via alternative channels, such as marriage markets, which, to date, have been largely ignored in the literature. In this paper, we show that sanitation matters in marriage markets — the Indian Total Sanitation Campaign (TSC) changed marriage market outcomes. To show this, we exploit quasi-random variation from the TSC that shifted the distribution of households with sanitation, and thus the incentives of men and women to sort into marriage, or not. We show that exposure to a high TSC district had a significant impact on both the marital surplus and the sorting into marriages by men and women. The analysis relies on reduced-form techniques and a structural approach. Both approaches exploit information on marriages – including the chosen living arrangement of couples – across districts and time. Identification is achieved through a difference-in-differences approach in a multi-market framework. To conduct a structural policy analysis, we develop an equilibrium model of the Indian marriage market à la Choo and Siow (2006), where the choice of future spouse and sanitation within the marriage is considered jointly. The determinants of the marriage surplus include the presence of sanitation and socio-economic spousal characteristics, such as wealth.

Model estimates enable us to demonstrate the impact of the TSC policy on marriage markets. First, we document a significant increase in the marital surplus due to high TSC exposure, i.e., a decrease in the price of sanitation, a household public good, increases the gains from the marriage that a couple receives relative to singlehood. These gains make it more attractive to be in a marriage with sanitation in the arrangement, and, in line, we see a change in marital behavior away from singlehood. Second, the change in marital sorting resulted in a reallocation of gains across matched types. Specifically, the results display a marked gender asymmetry with an increase in marital surplus among matches where men are wealthier than their spouses and a decrease in surplus where the wife is wealthier. Lastly, the policy also redistributed gains within a marriage. On average, these gains are redistributed away from women. We demonstrate this by analyzing TSC’s impact on the wife’s surplus share, which shows that the division of gains was not necessarily equal among partners.

While other evaluations of the TSC tend to focus on the policy’s positive impacts on sanitation uptake, our paper draws attention to an important indirect effect that is potentially present in other such female-focused policies, including the “No Toilet, No Bride” campaign, but also in other female-focused interventions. For example, Angelucci and Bennett (2021) finds that removing HIV information asymmetry through high-frequency testing leads to accelerated marriages. We argue that in order to obtain a comprehensive picture of gender-focused policies, including costs and benefits, their interaction with marriage markets should not be ignored.

The insights derived from our empirical analysis further highlight interesting avenues for future research that are beyond the scope of this paper. Most notably, the importance of sanitation in the marriage market determined by the magnitude of unobserved heterogeneity raises interesting questions regarding the presence and nature of frictions specifically in the marriage market. Even though the transferable utility assumption limits our ability to explore such questions, our results emphasize a promising avenue to be explored in future research. In addition, while the marriage matching function in our analysis is static, it may be reasonable to expect additional aspects of marital behavior, in response to policy exposure, to be dynamic e.g., marriage delay. Moreover, this response may differ across men and women. In such a case, we would need a dynamic marriage matching function. While a dynamic matching model lies beyond the primary focus of this paper, an important contribution of this paper is to provide a rigorous structural foundation that can be extended to explore resulting dynamics from the TSC. In summary, our analysis emphasizes the importance of accounting for general equilibrium effects, which necessitates going beyond reduced-form methods and yet has been largely omitted from the policy discourse within the literature at the intersection of sanitation and marriage markets.

## Declaration of Competing Interest

The authors declare that they have no known competing financial interests or personal relationships that could have appeared to influence the work reported in this paper.

## Data Availability

The data that has been used is publicly available. Code will be made available upon request

## Appendix A Supplementary data

MMC S1 Online appendices A, B, and C.

## Footnotes

1 Several other programs are known to have effects on marriage markets — for instance, the introduction of divorce laws (Reynoso, 2018, Greenwood et al., 2016, Fernández and Wong, 2017, Rasul, 2006), the incarceration of offenders (Charles and Luoh, 2010), compulsory schooling (Hener and Wilson, 2018), and school construction (André and Dupraz, 2019, Zha, 2019).

2 The importance of marriage markets and changes in sorting was first emphasized by Lundberg and Pollak (1996).

3 The successor policy was the Nirmal Bharat Abhiyan (NBA), implemented from 2012, which in 2014 evolved into the current Swachh Bharat Mission.

4 A GP is the smallest administrative unit in India and is charged with the delivery of a number of programs, including the TSC.

5 The IEC approach of the TSC aligned closely with the Community-Led Total Sanitation (CLTS), adopted by more than 25 governments around the world as part of their sanitation strategy (Zuin et al., 2019).

6 The awareness creation approach used in the program CLTS was shown to be effective at increasing sanitation investment in, for example, Tanzania (Briceno et al., 2017), Mali (Pickering et al., 2015), Nigeria (Abramovsky et al., 2023), Ghana (Crocker et al., 2016), and Ethiopia (Crocker, 2016). A study by Guiteras et al. (2015) demonstrates the effectiveness of CLTS alongside subsidy provision in the context of Bangladesh.

7 The first indicator is an input (% of TSC budget spent) for which the maximum score is 5; the next two are outputs (% of household toilet targets achieved, 15 scores, and % of school sanitation target achieved ten scores); there are three process indicators with a maximum of ten scores for each (financial efficiency (cost per NGP community), the average population per community (gram panchayat), the success rate of NGP applications; and two outcomes indicators (number of NGP panchayats, 30 scores, and % of NGP panchayats, ten scores.

8 There is no information on the Grand Score in the first wave for all districts in Chandigarh, Delhi, Daman and Diu, Dagra, and Nagar Haveli, and some districts in Uttar Pradesh (1), West Bengal (4), Orissa (1), Madhya Pradesh (1), Maharashtra (4), Andhra Pradesh (1), Karnataka (2), and Tamil Nadu (2).

9 The practice is closely linked with endogamy (marriage within one’s own caste group although outside one’s own “gotra”, i.e., clan), which we will discuss further below.

10 Assortative matching based on characteristics such as income, education, physical characteristics, and age have been similarly established in other contexts (see, for example, Chiappori et al., 2017, Chiappori et al., 2018, Becker, 2009, Grossbard, 1993, Choo and Siow, 2006, Pencavel, 1998, Chiappori et al., 2012 and Hitsch et al. (2010)). Adams-Prassl and Andrew (2019), for example, highlight how marriage market returns provide the primary motivation for investing in one’s daughter’s college education in India, which supports other literature that demonstrates positive returns to (female) education on the marriage market (Lafortune, 2013, Attanasio and Kaufmann, 2017). In our context, education positively and strongly correlates with wealth (correlation coefficient of 0.50). Our empirical analysis uses education as a control variable.

11 The assets included among the components are a bicycle, sewing machine, generator set, grinder, motorcycle, TV, air cooler, clock, electric fan, chair/table, cot, telephone, cell phone, refrigerator, pressure cooker, car, air conditioner, washing machine, computer, and credit card. Further details are available in Appendix B.

12 Figure A2 presents a graphical representation of the matching patterns from the perspective of women.

13 Figure A1 presents an alternative view of the same phenomenon: the proportion of respondents who are married by age.

14 Gupta (2014) discusses how male marriage rates show greater variation by region and over time than female marriage rates and depend on the availability of brides.

15 While we are not aware of any study making use of exogenous variation in sex ratios in India, studies analyzing the co-existence of sex selection and dowries in India (Borker et al., 2019, Murthi et al., 1995, Das Gupta et al., 2003, Basu, 1999) provide evidence of a link between sex selection and marriage markets.

16 Another aspect to consider is the presence of dowries. Table A1 shows that more than a third of households in the sample report that “gifts” at marriage are standard in their context. Yet, the illegality of dowries in the Indian context makes accurate measurement a challenge. Moreover, Botticini and Siow (2003) warn against interpreting dowries as the sole form of transfer that clears the marriage market. As such, for our analysis, the dowry response can be thought of as being encapsulated within the market clearing transfer.

17 The corresponding matching matrices, which provide numbers underlying the percentages presented here, are provided in Table A6.

18 Figure A3 is the same as Fig. 6 but for women. Similar types of patterns, with same-wealth levels dominating matches, are found for them, both in high and low TSC districts.

19 Stopnitzky (2012) incorporates a Chiappori et al. (2009) framework to analyze pre-marital investment response in sanitation take-up under transferable utility and gender differences in preferences. In contrast, our analysis focuses on quantifying the relative attractiveness of sanitation in the marriage market. We model spousal and living arrangements as a simultaneous choice without a priori restrictions on the preference parameters across genders.

20 This is a standard assumption and widely employed within the marriage market literature to parameterize the distribution of the shocks; see, for example, Chiappori et al. (2017) and Choo and Siow (2006).

21 The difference-in-difference approach here is similar to CS. To implement their approach in Section 5, we assume that there are no time-varying effects that are district specific. This assumption rules out district-specific cultural change (over time) on the relevance of specific attributes in the match decision while allowing for a general time trend common to all districts.

22 Our approach is similar to the difference in difference estimator implemented by Choo and Siow (2006) to analyze the impact of the partial legalization of abortion in the US on the total gains to marriage.

23 Table A9 and A10 present the same set of results for districts with high and low sex ratios separately. The results are similar for both sets of markets.

24 The measures we use rely on consumption spending (expenditures on food, individual care articles, and children’s education) and women’s self-reported response to questions on household financial and non-financial decision making within the household.
