# Supplementary material for: Sanitation and marriage markets in India: Evidence from the Total Sanitation Campaign
Source: J Dev Econ. 2023 Jun;163:103092. doi: 10.1016/j.jdeveco.2023.103092 (PMC10273184; doi:10.1016/j.jdeveco.2023.103092)
Supplement: MMC S1 — Online appendices A, B, and C. [file mmc1.pdf]

Sanitation and marriage markets in India: Evidence from the Total Sanitation Campaign

Britta Augsburg, Juan P. Baquero, Sanghmitra Gautam, Paul Rodriguez-Lesmes

Online Appendix

A Descriptives

A.1 Figures

Figure A1: Fraction of married respondents

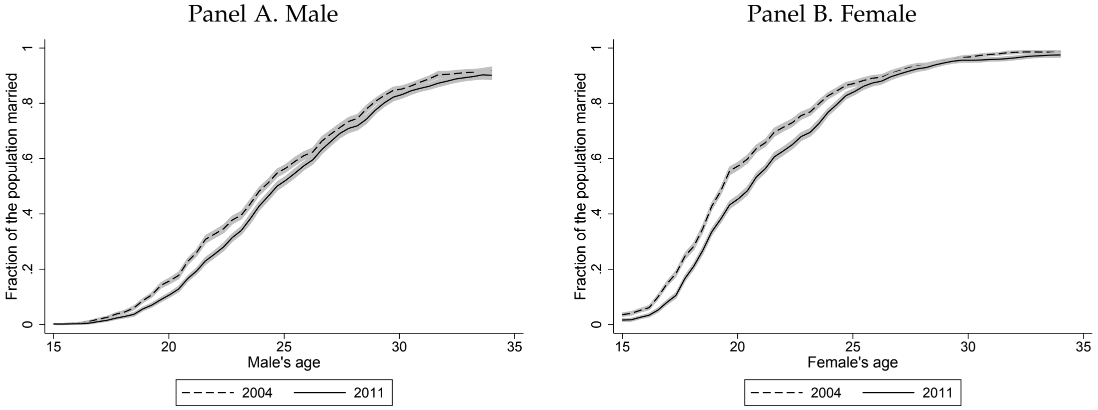

Note. The figure shows the fraction of the population married by gender. The fraction is conditional on age, gender and year of IHDS survey. Sample includes all men and women aged 15 to 34 years.

Figure A2: Marriage patterns over time (Women)

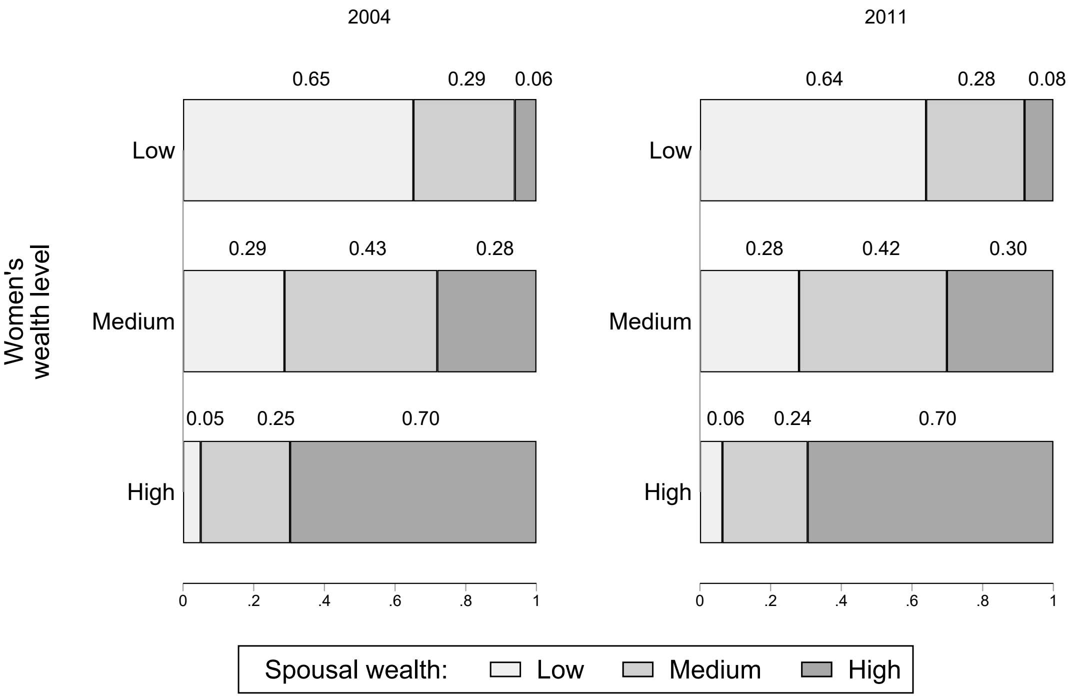

Note. The sample consists of households of all married women aged 15 to 34 at the time of the survey. 'High wealth' correspond to individuals whose asset index is above the 66th percentile of the entire country distribution per wave. Households below the 33th percentile cutoff are classified as having 'low wealth'. Between the two cutoffs, households are classified as 'medium wealth'. Proportions are computed using frequencies reported in Table A6.

Figure A3: Marriage patterns over time across TSC exposure groups (Women)

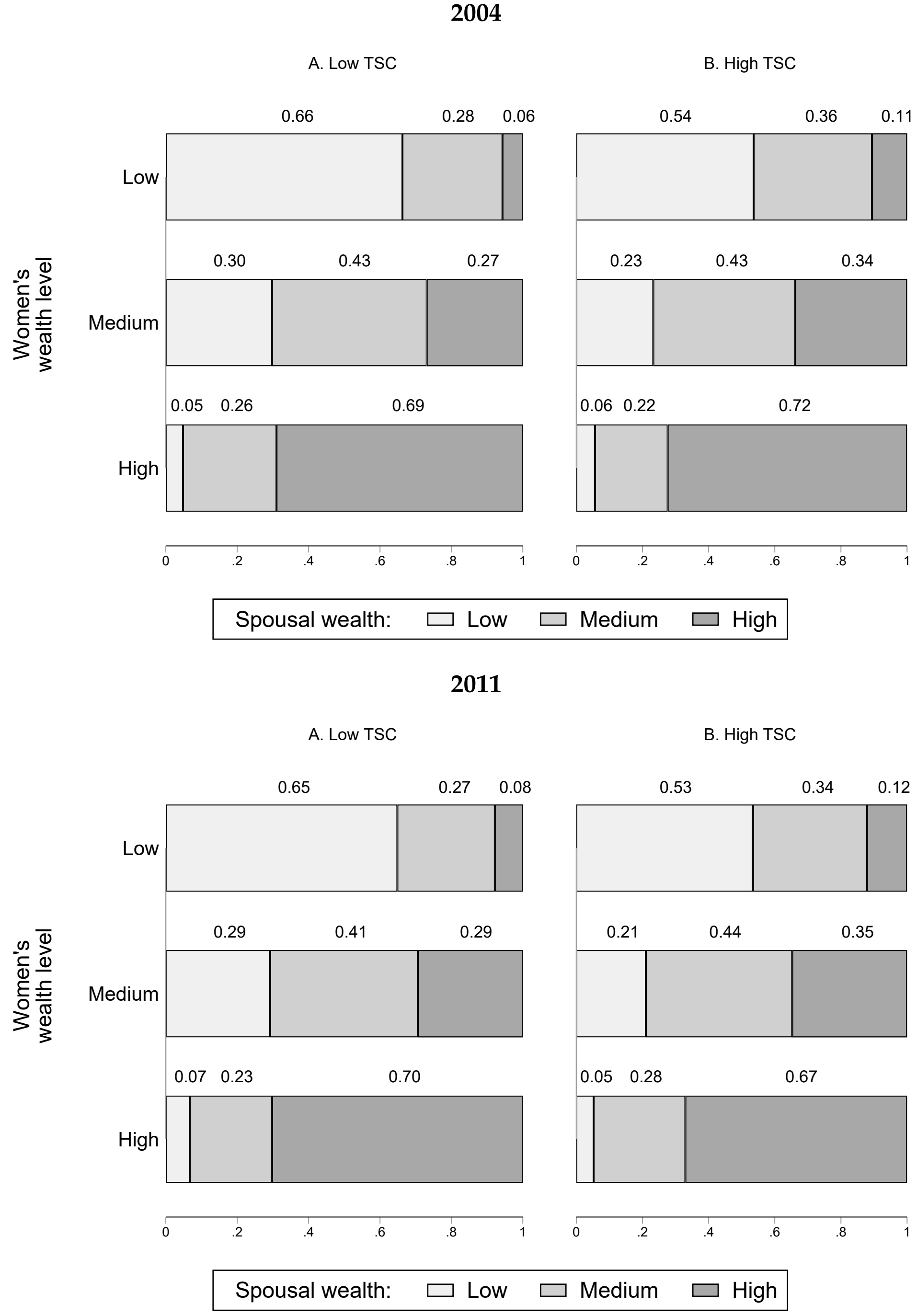

Note. The sample consists of households of all married women aged 15 to 34 at the time of the survey. The figure shows the marriage patterns of women over time across TSC exposure groups. High TSC corresponds to a grand score for implementation of at least 61, and Low TSC otherwise. The Grand Score per district is taken from WSP (2011). 'High wealth' correspond to individuals whose asset index is above the 66th percentile of the entire country distribution per wave. Households below the 33th percentile cutoff are classified as having 'low wealth'. Between the two cutoffs, households are classified as 'medium wealth'. Proportions are computed using frequencies reported in Table A6.

A.2 Tables

Table A1: Descriptive statistics, IHDS sample

	2004	2011
**Demographics:**		
Age at marriage (women):		
p25	15.0	16.0
p50	17.0	18.0
p75	19.0	20.0
mean	17.1	18.0
Age gap (male-female):		
p25	0.0	0.0
p50	1.0	1.0
p75	4.0	3.0
mean	2.1	1.8
**Marriage characteristics:**		
Newly-weds living with groom's parents	0.924	0.974
Wife from same village as groom	0.119	0.105
Hours to wife's natal home (mean)	3.287	3.065
Inter-caste marriage	0.049	0.060
**Marriage associated costs:**		
Gifts occurrence (0: never; 1: usually)	0.448	0.364
Share of household income spent by the groom	1.7	1.9
Share of household income spent by the bride	2.6	2.9

Note. Own calculations using data from the IHDS waves 2004 and 2011. The sample includes men and women aged 15 to 34 years and married at the time of the survey.

Table A2: Descriptive statistics by TSC groups (IHDS 2004)

	Low TSC	High TSC
**Demographics:**		
Age at marriage (women):		
p25	15.0	16.0
p50	17.0	18.0
p75	19.0	20.0
mean	17.0	18.0
Age gap (male-female):		
p25	0.0	0.0
p50	1.0	0.0
p75	3.0	4.0
mean	2.0	2.3
**Marriage characteristics:**		
Newly-weds living with groom's parents	0.924	0.924
Wife from same village as groom	0.106	0.181
Hours to wife's natal home (mean)	3.293	3.278
Inter-caste marriage	0.047	0.056
**Marriage associated costs:**		
Gifts occurrence (0: never 1: usually)	0.463	0.376
Share of household income spent by the groom	1.76	1.38
Share of household income spent by the bride	2.67	2.51
**Caste:**		
Brahmin	0.054	0.044
High caste	0.134	0.204
Other backward caste	0.353	0.309
Dalit	0.212	0.233
Adivasi	0.091	0.025
Muslim	0.138	0.129
Sikh, Jain or Christian	0.018	0.056

Note. Own calculations using data from the IHDS wave 2004. The table presents descriptive statistics by TSC groups in 2004. Low TSC corresponds to districts with a Grand Score for implementation below 61, and high TSC to those with a number of 61 or above. The Grand Score per district is taken from WSP (2011).

Table A3: Sensitivity checks: Cut-off definitions

		Sanitation take-up							
**Panel A: High sex ratio cut-off**		985	987	989	991	993	995	997	**999**
Oldest marriageable son	All	0.061*	0.061*	0.061*	0.061*	0.061*	0.061*	0.061*	0.061*
		(0.032)	(0.032)	(0.032)	(0.032)	(0.032)	(0.032)	(0.032)	(0.032)
	HSR	−0.056	−0.054	−0.052	−0.070*	−0.065	−0.051	−0.054	−0.047
		(0.036)	(0.037)	(0.038)	(0.039)	(0.042)	(0.045)	(0.047)	(0.049)
	LSR	0.164***	0.143***	0.142***	0.137***	0.140***	0.139***	0.138***	0.132***
		(0.041)	(0.041)	(0.041)	(0.039)	(0.039)	(0.039)	(0.038)	(0.038)
		1001	1003	1005	1007	1009	1011	1013	1015
Oldest marriageable son	All	0.061*	0.061*	0.061*	0.061*	0.061*	0.061*	0.061*	0.061*
		(0.032)	(0.032)	(0.032)	(0.032)	(0.032)	(0.032)	(0.032)	(0.032)
	HSR	−0.060	−0.057	−0.057	−0.045	−0.077*	−0.092**	−0.097**	−0.082*
		(0.044)	(0.045)	(0.045)	(0.046)	(0.041)	(0.041)	(0.045)	(0.049)
	LSR	0.132***	0.132***	0.130***	0.128***	0.131***	0.119***	0.117***	0.122***
		(0.038)	(0.038)	(0.038)	(0.037)	(0.037)	(0.037)	(0.037)	(0.037)

		Sanitation take-up							
**Panel B: TSC cut-off**		49	51	53	55	57	59	**61**	63
Oldest marriageable son	All	0.017	0.037	0.036	0.042	0.045	0.052*	0.061*	0.063*
		(0.026)	(0.025)	(0.026)	(0.028)	(0.029)	(0.030)	(0.032)	(0.033)
	HSR	−0.037	−0.051	−0.049	−0.049	−0.048	−0.048	−0.047	−0.047
		(0.052)	(0.050)	(0.050)	(0.050)	(0.049)	(0.049)	(0.049)	(0.049)
	LSR	0.059*	0.093***	0.091***	0.107***	0.109***	0.127***	0.132***	0.140***
		(0.032)	(0.030)	(0.031)	(0.033)	(0.035)	(0.037)	(0.038)	(0.041)
		65	67	69	71	73	75	77	79
Oldest marriageable son	All	0.072**	0.067*	0.071*	0.072*	0.079*	0.039	0.021	0.022
		(0.035)	(0.037)	(0.041)	(0.043)	(0.044)	(0.042)	(0.048)	(0.051)
	HSR	−0.044	−0.044	−0.032	−0.032	−0.023	−0.023	−0.054	−0.065
		(0.050)	(0.050)	(0.053)	(0.053)	(0.056)	(0.056)	(0.050)	(0.050)
	LSR	0.137***	0.138***	0.123**	0.129**	0.129**	0.083	0.084	0.089
		(0.041)	(0.046)	(0.050)	(0.054)	(0.054)	(0.054)	(0.069)	(0.073)

Note. Own calculations using data from the IHDS waves 2004 ($Post = 0$) and 2011 ($Post = 1$). The sample consists of households of all single and married males aged 15 to 34 at the survey time. This table presents sensitivity check to different classifications of the cut-off and reproduces the three coefficients $HighTSC \times Post$ of Table 1, Panel A includes various high-sex-ratio (HSR) and low-sex-ratio (LSR) classification by changing the cut-off (Table 1 uses 999), and Panel B includes various $HighTSC = 1$ and $HighTSC = 0$ classifications (Table 1 uses 61). The Grand Score per district is taken from WSP (2011). District-level sex ratio information was computed using data from the population census 2001 and 2011. Apart from the coefficients presented in the table, as controls we use the age and marital status of the individual for whom the household is in the sample; the wealth index of the household; household size; education level of the household head (no education [base], primary, incomplete secondary, secondary, and above secondary); caste (Brahmin [base], high caste, other backward caste, Dalit, Adivasi, Muslim, and Sikh - Jain - Christian); an indicator variable for being in a rural zone; and fixed effects at the district level. Standard errors clustered at the district level are shown in parentheses. Significance: *** p<0.01, ** p<0.05, * p<0.1

Table A4: TSC impact on sanitation for households with daughters

	Sanitation take-up		
	All	High sex ratio	Low sex ratio
	(1)	(2)	(3)
$HighTSC \times Post$	0.034	-0.080	0.086***
	(0.027)	(0.050)	(0.033)
Observations	10,433	2,618	7,815
R^2	0.493	0.525	0.489
Wald test (2)=(3): p-val		0.007	

Note. Own calculations using data from the IHDS waves 2004 ($Post = 0$) and 2011 ($Post = 1$). The table shows the TSC impact on sanitation for households with an oldest marriageable daughter. The sample consists of households of all single and married females aged 15 to 34 at the survey time. $HighTSC = 1$ corresponds to a Grand Score for implementation of 61 or above; and $HighTSC = 0$ otherwise. The Grand Score per district is taken from WSP (2011). The high sex ratio corresponds to districts with at least 999 women per 1000 men in the age range 15-34, while the low sex ratio is districts with less than 999. District level sex ratio information was computed using data from the population census 2001 and 2011. As controls, we use *Post*; the age and marital status of the individual for whom the household is in the sample; the wealth index of the household; household size; education level of the household head (no education [base], primary, incomplete secondary, secondary, and above secondary); caste (Brahmin [base], high caste, other backward caste, Dalit, Adivasi, Muslim, and Sikh - Jain - Christian); an indicator variable for the rural zone; and fixed effects at the district level. Standard errors clustered at the district level are shown in parentheses. A Wald test of equivalence of coefficients was performed by jointly estimating coefficients for columns 2 and 3 in a single regression, interacting all variables (including controls) with a dummy indicating whether the observation corresponds to a high sex ratio district or not. Significance: *** p<0.01, ** p<0.05, * p<0.1

Table A5: TSC impact on sanitation for households by sex ratio

	Sanitation take-up					
	Only son marriageable households			Only daughter marriageable households		
	All (1)	High sex ratio (2)	Low sex ratio (3)	All (4)	High sex ratio (5)	Low sex ratio (6)
$HighTSC \times Post$	0.061*	-0.047	0.132***	0.034	-0.080	0.086***
	(0.032)	(0.049)	(0.038)	(0.027)	(0.050)	(0.033)
Wald test (son vs. daughter) p-val						
(1) = (4)	0.159					
(2) = (5)		0.860				
(3) = (6)			0.095			
Observations	27,993	5,784	22,209	10,433	2,618	7,815
R^2	0.480	0.516	0.476	0.493	0.525	0.489

Note. Own calculations using data from the IHDS waves 2004 ($Post = 0$) and 2011 ($Post = 1$). The table shows the TSC impact on sanitation for households with marriageable males and females by sex ratio. The sample consists of households of all single and married males (females) aged 15 to 34 at the survey time. Households are classified according to the gender of the eldest offspring or the household head within such age range. $TSC = 1$ correspond to a grand score for implementation of 61 or above, and $HighTSC = 0$ to those with a score of 61 or above. The grand score per district is taken from WSP (2011). The high sex ratio corresponds to districts with at least 999 women per 1000 men in the age range 15-34, while the low sex ratio is districts with less than 999. District level sex ratio information was computed using data from the population census 2001 and 2011. As controls, we consider *Post*, the age and marital status of the individual for whom the household is in the sample; the wealth index of the household; household size; education level of the household head (no education [base], primary, incomplete secondary, secondary, and above secondary); caste (Brahmin [base], High caste, Other backward caste, Dalit, Adivasi, Muslim, and Sikh - Jain - Christian); an indicator variable for the rural zone; and fixed effects at the state level. Clustered at the district level standard errors in parentheses. The test of equivalence correspond to a Wald test of equivalence of coefficients that was performed by jointly estimating coefficients of columns 1 and 4 / 2 and 5 / 3 and 6 in a single regression, interacting all variables (including controls) with a dummy indicating whether the observation correspond to a son or a daughter. Significance: *** p<0.01, ** p<0.05, * p<0.1

Table A6: Matching matrices

Matrix A. 2004, Low TSC

		Women: Low	Women: Med	Women: High	Single men
Men	Low	3265	1064	136	5374
	Med	1380	1546	740	5412
	High	280	961	1949	5520
	Single women	2602	3265	3908	

Matrix B. 2004, High TSC

		Women: Low	Women: Med	Women: High	Single men
Men	Low	253	168	43	686
	Med	169	310	168	1217
	High	50	244	553	1740
	Single women	312	749	1063	

Matrix C. 2011, Low TSC

		Women: Low	Women: Med	Women: High	Single men
Men	Low	2808	999	164	5253
	Med	1182	1416	564	5165
	High	339	1003	1719	5091
	Single women	2971	3370	3812	

Matrix D. 2011, High TSC

		Women: Low	Women: Med	Women: High	Single men
Men	Low	189	121	38	489
	Med	122	255	202	1156
	High	43	200	488	1471
	Single women	222	501	1122	

Note. Own calculations using data from the IHDS waves 2004 (matrices A and B) and 2011 (matrices C and D). High TSC (matrices B and D) corresponds to a grand score for implementation of at least 61, and Low TSC otherwise (matrices A and C). The Grand Score per district is taken from WSP (2011). 'High wealth' correspond to individuals whose asset index is above the 66th percentile of the entire country distribution per wave. Households below the 33th percentile cutoff are classified as having 'low wealth'. Between the two cutoffs, households are classified as 'medium wealth'. Of each of the four matrices, the first three rows [columns] correspond to the wealth type of the husband I [wife J]. Then, each combination is the total number of respondents who matched for the types IJ. The last row [column] presents the frequencies of single women type J [men type I].

Table A7: Marital surplus by living arrangement

**Wife →**	Wealth type L	Wealth type M	Wealth type H
**Husband ↓**		$\hat{\pi}^{IJT}$ with toilet	
Wealth type L	-1.054	-2.533	-5.148
	(0.045)	(0.053)	(0.133)
Wealth type M	-1.747	-1.692	-2.532
	(0.031)	(0.021)	(0.034)
Wealth type H	-2.956	-1.657	-0.549
	(0.059)	(0.022)	(0.019)
**Husband ↓**		$\hat{\pi}^{IJN}$ without toilet	
Wealth type L	-0.364	-1.546	-3.769
	(0.018)	(0.043)	(0.137)
Wealth type M	-2.171	-1.633	-2.078
	(0.030)	(0.014)	(0.047)
Wealth type H	-4.513	-2.414	-1.435
	(0.073)	(0.025)	(0.031)
**Husband ↓**		$\hat{\pi}^{IJT} - \hat{\pi}^{IJN}$	
Wealth type L	-0.690	-0.987	-1.379
	[0.000]	[0.000]	[0.000]
Wealth type M	0.423	-0.058	-0.454
	[0.000]	[0.012]	[0.000]
Wealth type H	1.558	0.756	0.885
	[0.000]	[0.000]	[0.000]

Note. This table presents the marital surplus estimates for a given couple IJ in marriages with (top panel) and without (middle panel) a sanitation facility at home, with standard errors presented in parentheses. The bottom panel presents the difference in marital surplus $\hat{\pi}^{IJT} - \hat{\pi}^{IJN}$ and corresponding t-test p-value shown in square brackets. Wealth Types L, M, and H refer to low, medium and high wealth respectively. Standard errors, shown in parentheses, are clustered at the district level and computed using 1000 bootstrap replications.

Table A8: Aggregate TSC impact

	Martial Surplus	Surplus Share
	(1)	(2)
$HighTSC \times Post$	0.546***	-0.047
	(0.101)	(0.034)

Note. This table presents the TSC impact on the aggregate marital surplus and female surplus share estimates – with a weighted average taken across both living arrangements. The TSC policy exposure is defined by $HighTSC \times Post$ where $HighTSC = 1$ corresponds to a grand score for implementation of at least 61, and $HighTSC = 0$ otherwise and *Post* is indicator that take value 1 in the 2011 period. Standard errors, shown in parentheses, are computed using a bootstrap procedure with 1000 replications and are clustered at the district level. Significance: *** p<0.01, ** p<0.05, * p<0.1.

Table A9: TSC impact of marriage rate (shortage of women)

	Marriage rate	
	Men	Women
	(1)	(2)
Wealth type L	0.031***	0.061***
	(0.008)	(0.008)
Wealth type M	0.008	-0.006
	(0.007)	(0.007)
Wealth type H	0.003	-0.011
	(0.005)	(0.010)

Note. This table presents the TSC policy impact on the marriage rate of men (column 1) and women (column 2) in markets with a shortage of women, i.e., low sex ratio. Wealth Types L,M,H refer to low, medium, and high wealth, respectively. Each cell presents the difference-in-difference estimate. The base marriage rate among men is 0.442 (type L), 0.399 (type M), and 0.353 (type H). While the base marriage rate among women is 0.663 (type L), 0.535 (type M), and 0.359 (type H). Regression specification includes district and time fixed effects. Household level controls include the age of the man (or woman) and caste. Standard errors, shown in parentheses, are clustered at the district level and computed using 1000 bootstrap replications. Significance: *** $p<0.01$, ** $p<0.05$, * $p<0.1$.

Table A10: TSC impact on marriage rate (excess of women)

	Marriage rate	
	Men	Women
	(1)	(2)
Wealth type L	0.058***	0.135***
	(0.010)	(0.010)
Wealth type M	0.017**	0.005
	(0.008)	(0.009)
Wealth type H	0.001	0.005
	(0.010)	(0.009)

Note. This table presents the TSC policy impact on the marriage rate of men (column 1) and women (column 2) in markets with an excess of women, i.e., high sex ratio. Wealth Types L,M,H refer to low, medium, and high wealth, respectively. Each cell presents the difference-in-difference estimate. The base marriage rate among men is 0.406 (type L), 0.362 (type M), and 0.320 (type H). While the base marriage rate among women is 0.609 (type L), 0.475 (type M), and 0.317 (type H). Regression specification includes district and time fixed effects. Household level controls include the age of the man (or woman) and caste. Standard errors, shown in parentheses, are clustered at the district level and computed using 1000 bootstrap replications. Significance: *** $p<0.01$, ** $p<0.05$, * $p<0.1$.

Table A11: TSC impact on partners of men

**Wife** $\rightarrow$	Wealth type L	Wealth type M	Wealth type H
**Husband** $\downarrow$	TSC impact on $\sum_M \widehat{P}(J\,\&\,M\vert I)$		
Wealth type L	0.083***	-0.102***	0.024
	(0.015)	(0.015)	(0.041)
Wealth type M	-0.009	-0.101***	0.087***
	(0.019)	(0.009)	(0.016)
Wealth type H	0.009	-0.065***	-0.011
	(0.032)	(0.017)	(0.007)

Note. Men in rows and women in columns. This table presents the TSC policy impact on the spousal choice probabilities for men conditional on male type. The spousal choice probabilities are averaged across both type of living arrangements. Wealth Types L,M,H refer to low, medium, and high wealth, respectively. Each cell presents the difference-in-difference estimate. Regression specification includes district and time fixed effects. Household level controls include the age of the man and caste. Standard errors, shown in parentheses, are clustered at the district level and computed using 1000 bootstrap replications. Significance: *** p<0.01, ** p<0.05, * p<0.1.

Table A12: TSC impact on partners of women

**Husband** $\rightarrow$	Wealth type L	Wealth type M	Wealth type H
**Wife** $\downarrow$	TSC impact on $\sum_M \widehat{P}(I\,\&\,M\vert J)$		
Wealth type L	0.074***	0.019	0.036
	(0.020)	(0.015)	(0.036)
Wealth type M	-0.045**	-0.045***	0.095***
	(0.023)	(0.010)	(0.017)
Wealth type H	-0.077***	-0.004	0.031***
	(0.025)	(0.019)	(0.011)

Note. Women in rows and men in columns. This table presents the TSC policy impact on the spousal choice probabilities for women conditional on female type. The spousal choice probabilities are averaged across both type of living arrangements. Wealth Types L,M,H refer to low, medium, and high wealth, respectively. Each cell presents the difference-in-difference estimate. Regression specification includes district and time fixed effects. Household level controls include the age of the woman and caste. Standard errors, shown in parentheses, are clustered at the district level and computed using 1000 bootstrap replications. Significance: *** p<0.01, ** p<0.05, * p<0.1.

B Wealth

In this appendix, we describe the construction and imputation procedure adopted for the matching attribute wealth.

Construction. The wealth index, defined at the household level, is based on a principal component analysis (PCA) of self-reported assets. The IHDS household survey includes a comprehensive income and asset module identifying a wide array of household assets. In defining our wealth measure, we include information on the following listed assets: bicycle, sewing machine, generator set, grinder, motorcycle, TV, air cooler, clock, electric fan, chair/table, cot, telephone, cell phone, refrigerator, pressure cooker, car, air conditioner, washing machine, computer, and credit card.

The PCA is run independently for each year, and we keep the first component. The first component captures 27% of the cumulative variance in 2004 and 26% in 2011, with eigenvalues of 5.7 and 5.4, respectively. Table B1 presents the average ownership of each asset and the corresponding eigenvectors. Rather than using standardized versions of each variable for computing the score (the sum of the corresponding eigenvector entry times the value of the variables), we use the binary variables. In this way, we obtain an index where 0 represents having none of the listed assets. Only 0.1% of the observations correspond to a 0.

The PCA produces an assets index estimate for each household in our sample. We use the estimated asset index to construct our main matching attribute of wealth types for men and women in the sample. Given the patrilocal nature of our context, both the asset index and the corresponding wealth type are observed for the men in our sample. In contrast, a similar asset measure is not observable for all the women in our sample. Specifically, for married women, we observe an asset index that corresponds to their husband's household index measure, while for single women, we observe an asset index that corresponds to their paternal home asset measure. To proceed, we use the household asset index estimate to assign a wealth type for all men, married and single, in our sample. Similarly, to characterize wealth types for single women, we use information from the parental household asset index. Lastly, to characterize wealth types for married women, we impute asset index values using information from households with single women. Assets are imputed using a linear model. Details of the procedure are described below.

Imputation. In order to obtain the asset index for women, we follow a two-step procedure. First, using the sample of single women in our sample, we run a regression of family household wealth on basic individual-level characteristics of women. The model includes age, age squared, years of education, years

of education squared, literacy, English knowledge, caste, religion, and district fixed effects. We use the estimated model to compute an asset value as a function of individual characteristics. In the second step, computed values from the first step are imputed as family wealth measures for the married women in the sample.

Results from the first step are shown in Table B2. In particular, it presents the coefficient estimates of the wealth regressions for both single men and single women. Estimates are done independently for each survey round of the IHDS. Columns 1 and 3 report coefficients for each year using the sample of single women. While columns 2 and 4 report coefficients for each year using the sample of single men. While we do not impute asset values for men, we follow the same procedure for men as a robustness measure to assess the validity of the exercise. In the second step, we use the estimates from Table B2 to predict the asset index value for married women and men separately. The imputed asset index distributions for women are shown in Panel B of Figure 2 Panel B for each survey year, while Panel A of Figure 2 plots the computed asset index measure that is observed for all men in our sample.

Robustness. The imputation procedure for a woman's wealth measure does not allow for any direct robustness checks. However, we are able to use the sample of single men to construct a similar imputed value for their male married counterparts. In what follows, we lay out a series of robustness checks analyzing the difference between the observed and imputed asset index values for married men.

Figure B1 presents a scatter plot of the observed and imputed asset index values for married men. On the x-axis, we plot the observed asset value for married men. The imputed asset value on the y-axis is computed using estimates from the first step. The diagonal marks the 45-degree line. Panel (a) shows a correlation coefficient of 0.65 for the 2004 sample, and panel (b) shows a correlation coefficient of 0.63 for the 2011 sample.

Table B1: Assets means and eigenvectors

	2004		2011	
	(1) Proportion that owns	(2) Eigenvector 1	(3) Proportion that owns	(4) Eigenvector 1
Cycle/bicycle	0.550	0.038	0.544	0.026
Sewing machine	0.266	0.208	0.258	0.203
Generator set	0.014	0.118	0.020	0.121
Mixer/grinder	0.257	0.296	0.334	0.289
Motor cycle	0.187	0.279	0.286	0.278
Black & white TV	0.260	0.014	0.051	-0.008
Colour TV	0.297	0.312	0.611	0.302
Air cooler	0.129	0.240	0.168	0.235
Clock/watch	0.853	0.160	0.858	0.214
Electric fan	0.641	0.249	0.750	0.267
Chair/table	0.687	0.225	0.776	0.235
Cot	0.848	0.119	0.894	0.095
Telephone	0.170	0.286	0.081	0.196
Cell phone	0.087	0.247	0.802	0.218
Refrigerator	0.177	0.317	0.274	0.316
Pressure cooker	0.445	0.283	0.540	0.297
Car	0.021	0.175	0.049	0.183
Air conditioner	0.006	0.133	0.019	0.149
Washing machine	0.044	0.230	0.094	0.245
Computer	0.013	0.151	0.067	0.220
Credit card	0.015	0.143	0.030	0.128

Note. Eigenvectors correspond to the first principal component after a principal component analysis is performed separately for each IHDS wave.

Table B2: Assets index as a function of individual characteristics

	Asset index			
	2004		2011	
	(1) Female	(2) Male	(3) Female	(4) Male
Age	0.027	-0.023*	-0.03	-0.014
	(0.016)	(0.013)	(0.023)	(0.015)
Age sq / 100	-0.040	0.078**	0.106**	0.060*
	(0.036)	(0.030)	(0.051)	(0.034)
Years of education	0.045***	0.038***	0.008	0.039**
	(0.013)	(0.012)	(0.020)	(0.015)
Years of education Sq	0.002***	0.002***	0.003***	0.001
	(0.001)	(0.001)	(0.001)	(0.001)
**English knowledge**				
Little	0.199***	0.178***	0.234***	0.194***
	(0.036)	(0.030)	(0.020)	(0.029)
Fluent	0.691***	0.498***	0.511***	0.474***
	(0.074)	(0.082)	(0.040)	(0.058)
Literacy	-0.102*	-0.097**	0.084	-0.031
	(0.057)	(0.040)	(0.089)	(0.039)
**Caste & religion**				
High caste	-0.101**	-0.099	-0.119*	-0.014
	(0.048)	(0.083)	(0.060)	(0.063)
Other backward castes	-0.366***	-0.354***	-0.366***	-0.269***
	(0.044)	(0.058)	(0.082)	(0.050)
Dalit	-0.479***	-0.514***	-0.513***	-0.407***
	(0.055)	(0.080)	(0.078)	(0.060)
Adivasi	-0.644***	-0.647***	-0.679***	-0.622***
	(0.064)	(0.091)	(0.052)	(0.077)
Muslim	-0.180***	-0.148	-0.172**	-0.027
	(0.057)	(0.123)	(0.071)	(0.122)
Sikh, Jain, or Christian	-0.139	0.027	-0.125	0.121
	(0.107)	(0.156)	(0.117)	(0.105)
Observations	11,933	19,441	12,027	18,660
R^2	0.45	0.39	0.44	0.4

Note. All regression specifications include district fixed effects and are weighted using survey design weights. Standard errors in parentheses are clustered at the district level. Categorical variables are defined as follows: English knowledge takes three values with None as the base category followed by little knowledge and being fluent. Literacy is an indicator taking value 1 if the individual is deemed literate, with illiterate as the base category. Caste and religion, a composite variable in the IHDS survey data, provides a summary measure of the caste and religious identity of individuals. Caste and Religion takess seven values with Brahmin being the base category. Significance: * p<0.10, ** p<0.05, *** p<0.01. *Sample:* Unmarried men and women.

Figure B1: Observed and imputed asset index values for men

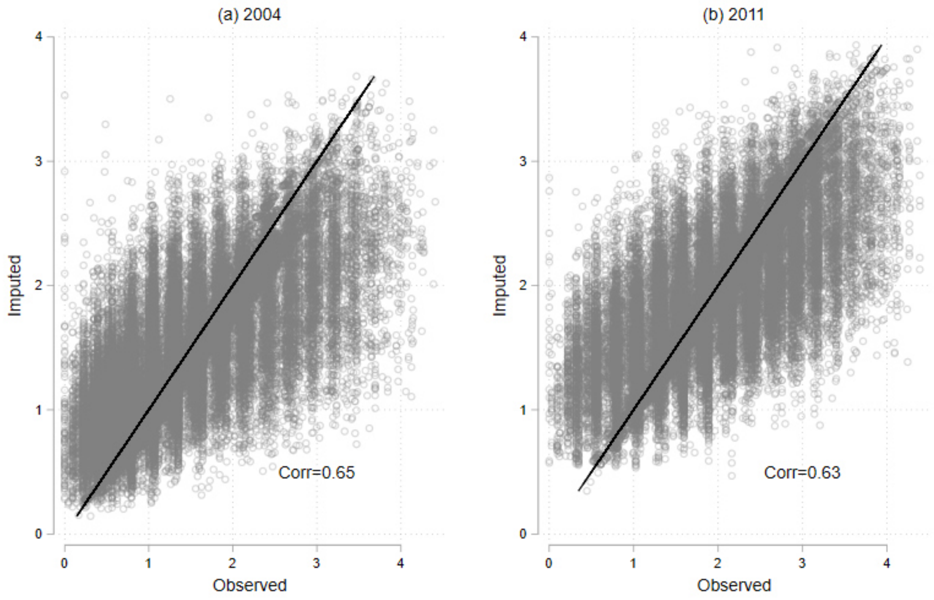

Note. Each circle represents an individual married male. The dark grey solid line corresponds to the 45 degree line. Panel (a) shows the scatter plot for the 2004 sample. Panel (b) shows the scatter plot for the 2011 sample. The correlation coefficient for the Panel (a) sample is 0.65 (p-value < 0.001). The correlation coefficient for the panel (a) sample is 0.63 (p-value < 0.001).

To examine the extent of deviations, we measure the difference between the imputed asset index and the corresponding observed asset index for each married male. Figure B2 presents the density of the resultant difference in values. As before, we present the difference measure for each of the survey years. For each year, these differences are standardized using the respective standard deviation of the variable capturing the different asset values (0.60 in 2004, 0.64 in 2011). For the 2004 sample, Figure B2 shows that the actual asset index tends to be lower than the imputed measure (mean: -0.161, p-value<0.001). We observe a similar pattern for the 2011 sample, though to a lesser extent (mean: -0.035, p-value<0.001).

Figure B2: Difference in asset value (observed - imputed) for men

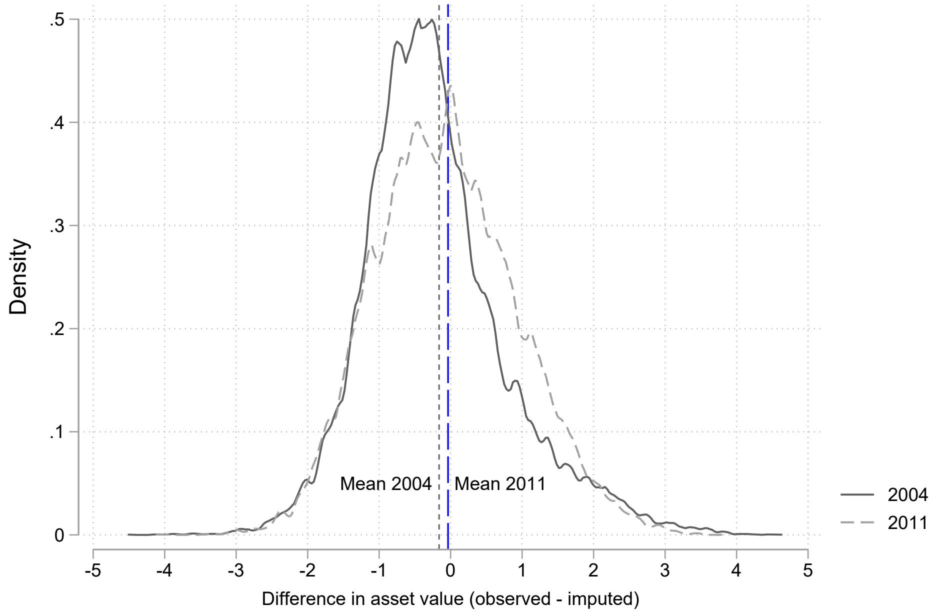

Note. This graph shows the distribution of the difference in asset value for each of the survey years. The difference is computed using the observed and imputed asset value for married men in our sample.

Lastly, we examine whether the difference between the observed and imputed values varies in a systematic way across other marriage features at the market and individual levels. Panel (a) of Figure B3 shows a box plot of the differences in asset value across the composite caste religion groups in our sample. Similarly, Panel (b) shows the differences across age categories. Differences in the observed and imputed asset value across education categories are included in panel (c).

Figure B3: Box plots of the difference in asset value (observed - imputed) for men

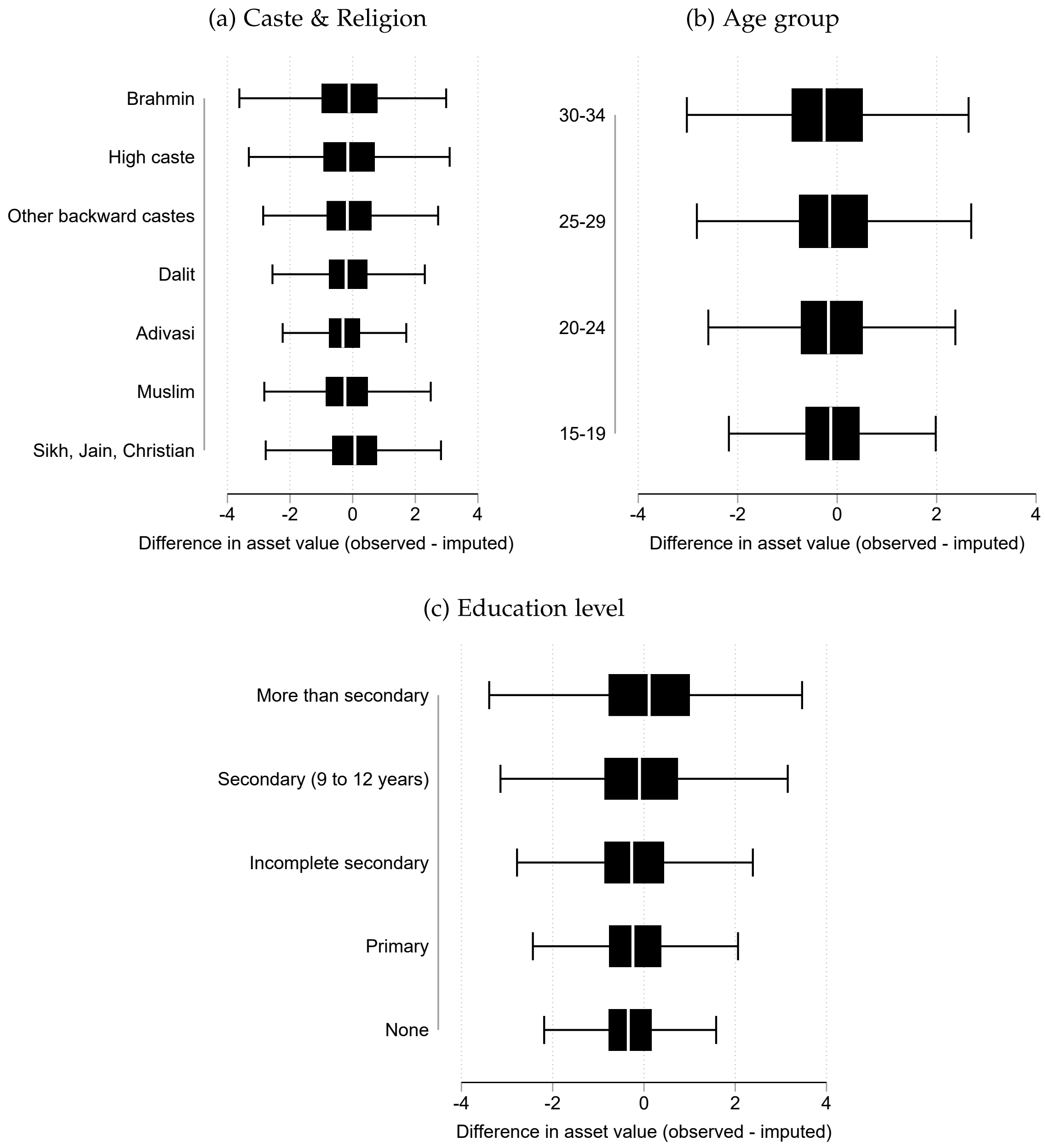

Note. Panel (a) presents box plots by composite caste and religion categories of the difference between observed and imputed asset values males. Outside values are omitted. Panel (b) considers age groups, and Panel (c) education levels.

C Corroborative evidence on surplus share

Here we provide indirect evidence that the impacts on expected surplus share discussed in Section 5.2 are likely reflecting similar impacts on gender-specific expenditure shares. We consider two sets of measures of intra-household allocation of residual resources. Our analysis in this section considers a sample of all households that includes a married couple where the main woman was aged 15 to 34 at the time of the survey and who had been married within the past six years (the span between the two IHDS waves).

The first measure is consumption spending, specifically expenditures on food, individual care articles (toothpaste, hair oil, shaving blades, etc.), and children's education (school fees, private tuition fees, school books, stationery, and other educational articles). Food, personal care, and education are household spending categories that typically increase with women's bargaining power (Brown et al., 1994; Masterson, 2012; Thomas, 1990). Using the IHDS data, we construct the share of food, the share of individual care articles (toothpaste, hair oil, shaving blades, etc.), and the share of children's education in household spending. A value of 0 reflects a household that does not spend on such items; a value of 1 indicates that the entire expenditure is represented by the specific category.

For our second set of measures, we construct two 'decision-making indices' based on women's self reported response to questions on household decisions. These types of questions are available in the women's questionnaire of the IHDS (gender relations module 16) and have been previously used to measure similar concepts (Heath and Tan, 2020). Specifically, we use two sets of questions, one relating to financial decisions and the other to non-financial ones. Questions on financial decisions ask who in the household has the final say on (i) buying an expensive item such as a TV or a fridge and (ii) doing food shopping. Non-financial decisions ask about who decides (i) what to cook daily, (ii) how many children to have, and (iii) what to do if a child falls sick. Including both types of questions in our index is in line with recent literature that shows that women's decision-making within the household varies with the decision being made (e.g., Peterman et al. (2021)). We generate a decision-making index by averaging responses for each of the decisions taken. The responses are re-coded to range from 0 (woman reports not to be involved in any of the decisions) to 1 (woman takes all decisions by herself only). Intermediate values are 0.33 (the woman is involved, together with other household members, other than her husband) and 0.66 (woman and spouse are involved in decision-making).[24]

Results are presented in Table C1. Each row refers to a different outcome. Column 1 displays the mean for the outcome in 2011 in areas with low TSC exposures ($TSC = 0$). Column 2 presents the regression estimates of the impact of TSC on the respective outcome, coefficient γ in Equation 1. Columns 3 and 4 show the impacts by low and high wealth, respectively.

The table shows that in areas of low TSC exposure, in 2011, the budget share devoted to food is close to 50%, a common finding in the country (FAO and The World Bank, 2019), and expenditures on personal

[24]For the question on food and vegetable shopping, the question does not directly mention the husband but 'adult men'. For this question, we assume that such an answer involves the husband.

care items and education are low at 1.6% and 2% respectively. Given that these are newly married couples, the small budget share on education is expected since any children born into the marriage would likely be under 6 years of age. In terms of the indices of participation in decision-making, we see that women are unlikely to take part in financial decisions (mean below 0.33), while participation in non-financial decisions is more common (mean above 0.33).

We find that, on average, impacts on these outcomes are (while negative for all but food consumption) small in magnitude and statistically insignificant (column 2). Among households of low wealth (column 3), the negative impacts on personal care items and children's education become significant and large in magnitude (roughly half of the average value reported in column 1). For the highest wealth tertile (column 4), the results are still negative but more imprecise.

Table C1: TSC impact on proxies of women's control over household resources

	Mean	$HighTSC \times Post$		
	(1)	(2)	(3)	(4)
Outcome	$Post = 1, HighTSC = 0$	All	Low wealth	High wealth
Household budget share:				
Food consumption	0.488	0.002	0.001	-0.003
		(0.011)	(0.022)	(0.015)
Personal care items	0.016	-0.001	-0.007**	-0.001
		(0.002)	(0.003)	(0.002)
Children's education	0.02	-0.004	-0.008***	-0.009
		(0.004)	(0.002)	(0.010)
Women's decision-making indices:				
Financial index	0.163	-0.003	0.034	0.029
		(0.015)	(0.026)	(0.020)
Non-financial index	0.426	-0.035	-0.009	-0.055*
		(0.023)	(0.026)	(0.031)
Observations		7,990	2,792	2,537

Note. Own calculations using data from the IHDS waves 2004 ($Post = 0$) and 2011 ($Post = 1$). The sample consists of all households that included a married couple where the main woman was aged 15 to 34 at the time of the survey and who had been married for less than six years (the span between the two IHDS waves). *Children's education* includes tuition fees, books and materials. The *non-financial index* includes decisions about cooking, number of children, and what to do if a child falls sick, whereas the *financial index* covers decisions on buying expensive items and doing shopping. *High (low) wealth* corresponds to individuals whose asset index is above the 75th (below the 25th) percentile of the entire country distribution per wave. $HighTSC = 1$ corresponds to a Grand Score for TSC implementation of at least 61, and $HighTSC = 0$ otherwise, as taken from WSP (2011). The estimation includes the following controls: age and marital status of the respondent; the household's wealth index and size; education level of the household head (no education [base], primary, incomplete secondary, secondary, and above secondary); caste (Brahmin [base], high caste, other backward castes, Dalit, Adivasi, Muslim, and Sikh - Jain - Christian); whether the household is rural, or not; and fixed effects at the district level. Standard errors are clustered at the district level and shown in parentheses. Stars indicate significance levels: *** p<0.01, ** p<0.05, * p<0.1

To get an alternative view of the findings above, Figure C1 considers only data from 2011 and includes couples where spouses are aged 15 to 34 but may have been married for more than six years. Those longer marriages are less likely to have been formed during the high exposure time of the program. Panel (a) represents the women's decision index on non-financial choices. Panels (b) and (c) consider two of the budget shares, plotting shares. All the panels are plotted by year of marriage for couples living in areas of

low and high TSC exposure. In general, we see that budget shares on children's education and personal care are higher for couples that got married in earlier years. We observe that couples who have been married for more than six years present constant differences across cohorts of marriage, but those married in the last six years have consistently lower averages in high exposure districts, particularly for children's education.

Figure C1: Proxies of women's control over household resources

(a) Women's non-financial decision-making index

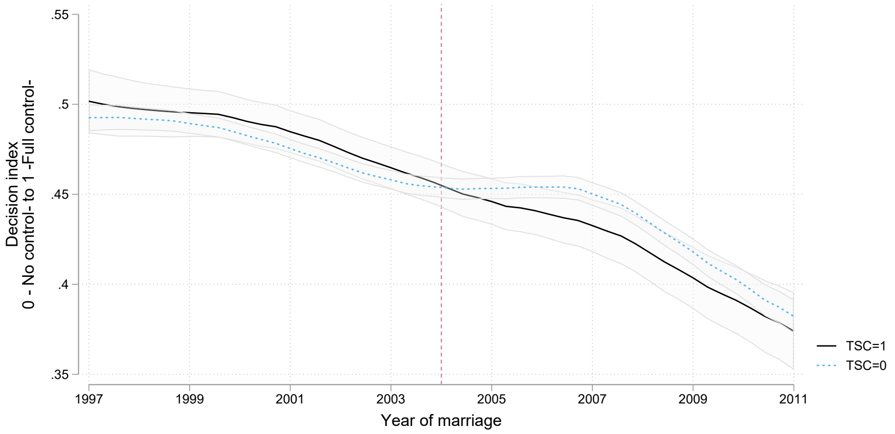

(b) Household budget allocated to children's education

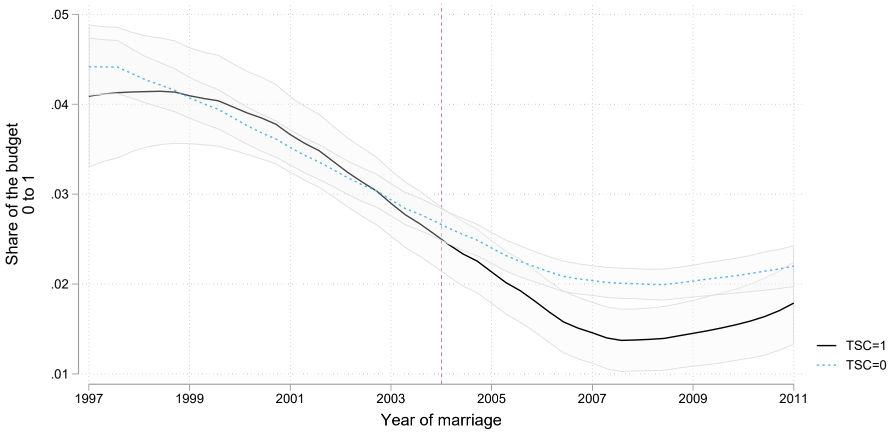

(c) Household budget allocated to personal care items

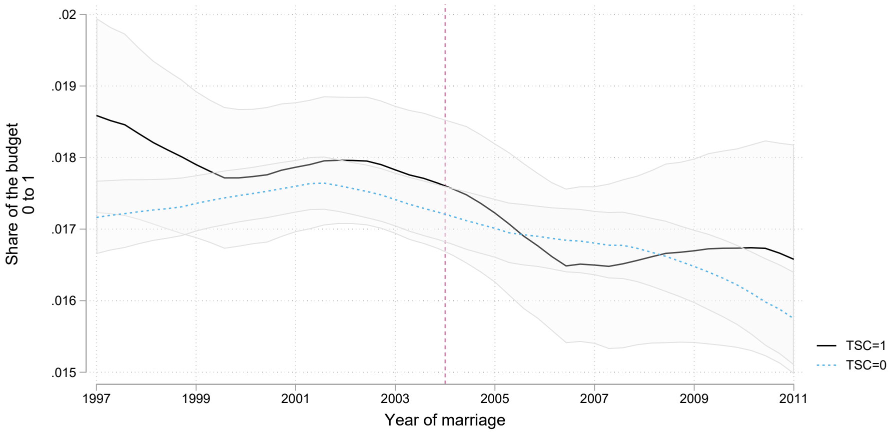

Note. This figure presents the relationship between various proxies of women's control over household resources according to the year of marriage (x axis). The sample consists of all households that included a married couple where the main woman was aged 15 to 34 at the time of the 2011 IHDS survey. The sample uses the IHDS 2011. Panel (a) Women's non-financial decision-making index (includes cooking, number of children, and whom they would marry). Panel (b) Share of the household budget allocated to children's education. Panel (c) Share of the household budget allocated to personal care items. Household budget shares are computed based on expenditures incurred at the time of survey.

References

Brown, L. R., Yohannes, Y., and Webb, P. (1994). Rural labor-intensive public works: Impacts of participation on preschooler nutrition: evidence from Niger. *American Journal of Agricultural Economics*, 76:1213–18.

FAO and The World Bank (2019). *Food Data Collection in Household Consumption and Expenditure Surveys – Guidelines for Low- and Middle-Income Countries*. Joint publication.

Heath, R. and Tan, X. (2020). Intrahousehold bargaining, female autonomy, and labor supply: Theory and evidence from india. *Journal of the European Economic Association*, 18(4):1928–1968.

Masterson, T. (2012). An empirical analysis of gender bias in education spending in paraguay. *World Development*, 40:583–593.

Peterman, A., Schwab, B., Roy, S., Hidrobo, M., and Gilligan, D. O. (2021). Measuring women's decisionmaking: Indicator choice and survey design experiments from cash and food transfer evaluations in Ecuador, Uganda and Yemen. *World Development*, 141:105387.

Thomas, D. (1990). Intra-household resource allocation: An inferential approach. *Journal of Human Resources*, 25:635—-664.

Water and Sanitation Program (WSP) (2011). A decade of the Total Sanitation Campaign: Rapid assessment of processes and outcomes. Volume 1: Main Report.
